# Lentiviral mediated delivery of CRISPR/Cas9 reduces intraocular pressure in a mouse model of myocilin glaucoma

**DOI:** 10.1038/s41598-024-57286-6

**Published:** 2024-03-23

**Authors:** Shruti V. Patil, Balasankara Reddy Kaipa, Sujata Ranshing, Yogapriya Sundaresan, J. Cameron Millar, Bhavani Nagarajan, Charles Kiehlbauch, Qihong Zhang, Ankur Jain, Charles C. Searby, Todd E. Scheetz, Abbot F. Clark, Val C. Sheffield, Gulab S. Zode

**Affiliations:** 1 Department of Pharmacology and Neuroscience, North Texas Eye Research Institute, University of North Texas Health Science Center at Fort Worth, Fort Worth, TX 76107 USA; 2 Department of Ophthalmology and Center for Translational Vision Research, University of California, 829 Health Sciences Rd, Irvine, CA 92617 USA; 3 Department of Pediatrics, University of Iowa, Iowa City, IA 52242 USA; 4 Department of Ophthalmology and Visual Sciences, University of Iowa, Iowa City, IA 52242 USA

**Keywords:** Myocilin-associated Glaucoma, Gene therapy, Genome editing for glaucoma, Trabecular meshwork, Intraocular pressure, Viral vectors, Lentiviral particles, ER stress, Hereditary eye disease, Molecular medicine

## Abstract

Mutations in myocilin (*MYOC*) are the leading known genetic cause of primary open-angle glaucoma, responsible for about 4% of all cases. Mutations in *MYOC* cause a gain-of-function phenotype in which mutant myocilin accumulates in the endoplasmic reticulum (ER) leading to ER stress and trabecular meshwork (TM) cell death. Therefore, knocking out myocilin at the genome level is an ideal strategy to permanently cure the disease. We have previously utilized CRISPR/Cas9 genome editing successfully to target *MYOC* using adenovirus 5 (Ad5). However, Ad5 is not a suitable vector for clinical use. Here, we sought to determine the efficacy of adeno-associated viruses (AAVs) and lentiviruses (LVs) to target the TM. First, we examined the TM tropism of single-stranded (ss) and self-complimentary (sc) AAV serotypes as well as LV expressing GFP via intravitreal (IVT) and intracameral (IC) injections. We observed that LV_*GFP* expression was more specific to the TM injected via the IVT route. IC injections of Trp-mutant scAAV2 showed a prominent expression of GFP in the TM. However, robust GFP expression was also observed in the ciliary body and retina. We next constructed lentiviral particles expressing Cas9 and guide RNA (gRNA) targeting *MYOC* (*crMYOC*) and transduction of TM cells stably expressing mutant myocilin with LV_cr*MYOC* significantly reduced myocilin accumulation and its associated chronic ER stress. A single IVT injection of LV_cr*MYOC* in *Tg-MYOC*^*Y437H*^ mice decreased myocilin accumulation in TM and reduced elevated IOP significantly. Together, our data indicates, LV_cr*MYOC* targets *MYOC* gene editing in TM and rescues a mouse model of myocilin-associated glaucoma.

## Introduction

Glaucoma is the second leading cause of irreversible blindness worldwide affecting about 70 million people^1–3^. Primary open angle glaucoma (POAG), the most common form of glaucoma is associated with progressive loss of retinal ganglion cell (RGC) axons and optic nerve degeneration^4–6^. Elevated intraocular pressure (IOP), a major risk factor for glaucoma is caused by increased resistance to aqueous humor (AH) outflow through the trabecular meshwork (TM)^7–9^. Despite TM being the major site of glaucomatous pathology^10^, mechanisms regulating outflow resistance in TM are poorly understood^11^. Glaucoma is a multi-factorial disease associated with genetic and environmental factors^12–15^. *MYOC* was the first glaucoma gene identified^16–18^ and is responsible for approximately 4% of POAG and most cases of juvenile-onset glaucoma (JOAG)^19–21^. *MYOC*-associated JOAG is often less-responsive to current medication since current treatments do not target the main pathology^22–25^. It is therefore critical to develop targeted therapies to prevent vision loss in young pediatric patients.

*MYOC* is abundantly expressed in TM cells and other ocular and non-ocular tissues^26–28^. However, the exact function of *MYOC* is still not clear, although there are suggestions that it may function as a matricellular protein^29–34^. Various studies have demonstrated that WT *MYOC* is not required for the regulation of IOP, however mutations in *MYOC* lead to a gain-of-function phenotype^35–40^. Overexpression or knockout of WT *MYOC* exhibited no ocular changes in mice^39,40^ indicating that the WT *MYOC* is not required for homeostasis of IOP. This is further supported by the findings that homozygous or heterozygous deletion of myocilin in humans is not associated with glaucoma^40–43^. Mutant *MYOC* forms detergent insoluble aggregates and accumulates in the endoplasmic reticulum (ER) causing ER stress^30,35,36,38,44,45^. The insufficiency of TM cells to resolve chronic ER stress results in cell death, leading to IOP elevation^44,46–48^. Since myocilin is not required for IOP regulation and mutant myocilin acquires toxic gain-of-function phenotype leading to TM cell death, knocking out myocilin at the genomic level becomes an attractive strategy for developing a novel therapy for *MYOC*-associated glaucoma.

The Clustered Regularly Interspaced Short Palindromic Repeats (CRISPR) in association with CRISPR-associated systems (Cas) is a powerful and widely used tool for genomic research^49,50^. It has two major components: an endonucleases enzyme Cas9 that cuts DNA and a gRNA that guides Cas9 to specific DNA sites. Together, they form a ribonucleoprotein (RNP) complex that can identify and cut DNA at the specific site. Once bound, Cas9 introduces a double strand break in the DNA. Gene knockouts can be generated due to indels incorporated by non-homologous end joining (NHEJ) or a homologous sequence can be simultaneously introduced for homology-directed repair (HDR)^49,50^.

Previously, our group has demonstrated the successful gene editing of *MYOC* using the CRISPR/Cas9 system in mice and human donor eyes^51^. In this study, the knockout of *MYOC* was targeted by designing gRNA targeting exon 1. The Cas9 + guide RNA was delivered using the adenovirus (Ad)-5, which has specific tropism toward the TM^52^. Although, Ad5 is a highly efficient system, Ad5 is inflammatory and induces a strong immune response in transduced tissues^53^. Considering our goal of clinical development, we sought to investigate other viral vectors including adeno-associated viruses (AAVs) and lentiviral (LV) particles to deliver Cas9 targeting *MYOC* to TM in vitro and in vivo models^54,55^. These viruses hold potential for clinical application due to robust delivery with long-term transgene expression, efficient transduction in post-mitotic cells, low immunogenicity, and minimal toxicity^56,57^. In the present study, we first explored whether various AAVs or LV particles have specific tropism to TM in in vitro and in vivo models. We further examined whether selected AAV or LV expressing Cas9 and gRNA targeting *MYOC* (cr*MYOC*) reduce myocilin misfolding and rescue glaucomatous phenotypes in in vitro and in vivo models.

## Methods

### Viral vector constructs

AAV2, self-complementary AAV2 (scAAV2) and Trp-Mutant scAAV2 (scAAV2^Trp-Mut^) were selected for the study based on previous studies that show tropism toward the trabecular outflow pathway^58^. Ready to use AAV2, scAAV2 and ScAAV2^Trp-Mut^ expressing GFP under the control of the CMV promoter were purchased from the Viral Vector Core at the University of Florida, Gainesville, FL. LV expressing GFP under the control of the CMV promoter (LV_*GFP*) was purchased from Vector Builder, Inc (Product ID: LVMP-VB160109-10005).

Guide RNA (gRNA) targeting *MYOC* (GGCCTGCCTGGTGTGGGATG) published in the previous study, had the highest efficiency and selectivity in targeting human *MYOC*^51^. In our current study, this same gRNA was cloned with *spCas9* in the shuttle vector for generating LV constructs. LV particles expressing Cas9 + g*MYOC*, LV expressing GFP and LV expressing Cas9 + scrambled gRNA were manufactured by Vector Builder, Inc. The LV_*Cas9* + scrambled gRNA expresses spCas9 with non-specific gRNA sequence that does not target any genomic DNA. A different gRNA (GACCAGCTGGAAACCCAAACCA) was designed for cloning into ssAAV2 vectors using *saCas9* (AAV2_cr*MYOC*; Product ID: AAV2 MP (VB 200728-1179 bqW)) as the packaging capacity of AAV is comparatively small. The efficiency of this gRNA to selectively target human *MYOC* was found to be equivalently high. We have utilized AAV2 expressing an empty cassette as a control (AAV2_Null; Viral Gene Core, University of Iowa).

### Mouse husbandry

All mice were housed and bred in a research facility at the University of North Texas Health Science Center (UNTHSC, Fort Worth, TX, USA). Animals were fed standard chow ad libitum and housed in cages with dry bedding. The animals were maintained in a 12 h light:12 h dark cycle (lights on at 0630 h) under a controlled environment of 21–26 °C with 40–70% humidity. C57BL/6J (male) mice were obtained from the Jackson Laboratories (Bar Harbor, ME, USA). We have utilized *Tg-MYOC*^*Y437*^^*H*^ mice that express mutant *MYOC* and develop ocular hypertension by the age of 3-months as described previously^46,59,60^. *Tg-MYOC*^*Y437H*^ mice on a pure C57BL/6J strain were utilized for this study. These mice were genotyped by PCR using primers specific to human *MYOC* as described previously^46,59,60^. Animal studies were executed in agreement with the guidelines and regulations of the UNTHSC Institutional Animal Care and Use Committee (IACUC) and the ARVO Statement for the Use of Animals in Ophthalmic and Vision Research. This study is reported in accordance with ARRIVE guidelines (https://arriveguidelines.org). Experimental protocols were approved by UNTHSC IACUC and Biosafety office under the approved protocol. At the end of experiment, mice will be sacrified by CO_2_ inhalation followed by cervical dislocation.

### TM cell culture and in vitro transduction

Transformed TM3 cells were transfected with pDsRed2-*MYOC* plasmids to generate stable cells expressing WT or mutant (Y437H or G364V) *MYOC* using Lipofectamine 3000 transfection kit (Invitrogen, Life Technologies, Grand Island, NY, USA). These plasmids express *MYOC* tagged with DsRed at the C-terminus. The confluent transfected cells were then treated with G418 antibiotic (0.6 mg/mL; Gibco, Life Technologies, Grand Island, NY, USA) for 7–10 days and individual colonies were selected and expanded. The cells stably expressing DsRed-tagged *MYOC* (with or without mutations) were characterized as described previously^61^, and maintained in DMEM media (Sigma-Aldrich Corp, St. Louis, MO, USA)) supplemented with G418 antibiotics, 10% FBS (Gibco), and streptomycin (Gibco). For viral transduction, TM3 cells were plated at 30–40% confluency. The following day, cells were incubated with AAV (5000 MOI/mL) or LV (10 MOI/mL) in antibiotic free and low serum (6%) media. 30 h post viral treatment, cells were switched back to regular maintenance medium. Once confluent (at day 3 or 4 post-transduction), cells were later processed for DNA isolation, Western blotting, and immunostaining. Human primary TM cells (n = 2 strains) were grown to confluency in 12-well plates and treated with AAV2/2, AAV2/4, AAV2/5 and AAV2/8 at multiplicities of infection (MOI) of 2.5 × 10^1^ to 2.5 × 10^3^ viral genomes (VG)/cell. GFP expression was examined by fluorescent microscopy after 72 h of transduction.

### Intraocular injections

Viral deliveries were performed via intravitreal (IVT) and intracameral (IC) routes. Mouse eyes were anesthetized before injections by topical administration of proparacaine HCl drops (0.5%) (Akorn Inc., Lake Forest, IL, USA). Both IVT and IC bolus injections were performed on mice anesthetized intranasally with isoflurane (2.5%; with 0.8 L/min oxygen). However, in case of slow-IC infusion protocol, mice were anesthetized using xylazine/ketamine (10/100 mg/kg; Vetus; Butler Animal Health Supply, Westbury, NY/Fort Dodge Animal Health, Fort Dodge, IA, USA) cocktail administered intraperitoneally. As required, additional one-quarter to one-half of the initial dose was provided for continuous maintenance of the surgical anesthetic state. LV particles (2.5 × 10^6^ TU/eyes and 2.5 μL/eye) or various AAV2 (2 × 10^10^ GC/eye) were injected via IVT or IC route. Hamilton’s (Reno, NV, USA) glass micro-syringe (10 μL capacity) attached with a 33 gauge 1-inch-long needle was used for IVT injections as described previously^62^. For IC route, mouse eyes were treated topically with 1% cyclopentolate (Mydriacyl, Alcon Laboratories, Fort Worth, TX) to dilate the pupils. Using the same micro-syringe system, the 33-gauge needle was inserted through the cornea 1–2 mm from the limbus, positioned parallel to the iris, and pushed towards the chamber angle opposite to the cannulation point. Care was taken to not touch the iris, corneal endothelium, or the anterior lens capsule. The viral solution was slowly released into the anterior chamber over a period of 30 s, after which the needle was kept inside for a further 1 min, before being rapidly withdrawn. For slow infusion, the glass micropipette system was loaded onto a micro-dialysis infusion pump (SP101I Syringe Pump; WPI) that delivered the viral solution at a flow rate of 0.083 μL/min over the course of 30 min (total volume delivered, 2.5 μL). A drop of filtered saline was also applied through this procedure to prevent corneal drying.

### IOP measurements

A TonoLab impact tonometer (Colonial Medical Supply, Londonderry, NH, USA) was used for IOP measurements on mice as previously described^63^. Baseline IOPs for C57BL/6J and *Tg-MYOC*^*Y437H*^ mice were measured during dark conditions (between 6:00 and 8:00 a.m.). The mice were anesthetized via intranasal isoflurane (2.5%; 0.8 L/min oxygen) delivery and readings were noted within 3 min of isoflurane influence to avoid any of its side effects on IOP^64^. Post-injections, IOPs were monitored weekly (daylight and dark) in a masked manner. The average value of six individual IOP readings were represented.

### Slit lamp imaging

A slit lamp (SL-D7, Topcon Corporation, Tokyo, Japan) was used to determine inflammation and ocular abnormalities in the anterior segment, including corneal edema, and photo-documented with a digital camera (DC-4; Topcon) as described earlier^46^.

### Histology and immunofluorescence staining

Following viral transduction, mice were euthanized at specified timepoints, and eyes were carefully enucleated and placed in 4% paraformaldehyde (PFA, Electron Microscopy Sciences, Hatfield, PA, USA) overnight at 4 °C. The next day, eyes were washed with 1 × PBS (Sigma-Aldrich) and cryopreserved using increasing concentration of sucrose (10% and 20%), followed by OCT compound embedding and sectioning. For hematoxylin and eosin (H&E) staining, the eyes were dehydrated in ethanol, and embedded in paraffin wax for sectioning. The paraffin-embedded mouse eyes were sectioned (sagittal) at 5 μm thickness, followed by deparaffinization in xylene, rehydration with gradual 5 min washes in each 100, 95, 70, and 50% ethanol solution and ending with a 10 min wash in 1 × PBS. These sections were later stained with H&E. The general morphology of the anterior segment was assessed including the TM structure at iridocorneal angle and corneal thickness by light microscopy. Images were captured using a Keyence microscope (Itasca, IL, USA).

The OCT-embedded sections from mouse eyes were incubated with 10% goat serum (EMD Millipore Corp) in 0.2% Triton X-100 (diluted in PBS; Fisher BioReagents, Fair Lawn, NJ, USA) for 2 h. For in vitro studies, TM cells were plated in 8-well chamber slides (Lab-Tek Nunc Brand Products, Rochester, NY, USA) and fixed with 4% PFA for 20 min, followed by PBS washes. Fixed cells or sections were then incubated with 10% goat serum in 0.1% Triton X-100 for 2 h. The slides were incubated with primary antibody (*MYOC*, catalog # 60357: Proteintech Group Inc, Rosemont, IL, USA; or GRP78, Catalog# ab21685: Abcam, Cambridge, MA, USA). The slides were washed 4 times with 1 × PBS before incubating with Alexa Fluor secondary antibody (1:500; Invitrogen, Life Technologies, Grand Island, NY, USA) at room temperature for 2 h. The slides were washed again and mounted with DAPI antifade mounting medium (Vectashield, Vector Laboratories Inc., Burlingame, CA, USA) as described previously^51,59,62,65^. For evaluating GFP expression in mice, the OCT sections were washed once with PBS and mounted with DAPI medium. Fluorescent images were captured, processed, and quantified using a Leica SP8 confocal microscope and LAS-X software (Leica Microsystems Inc., Buffalo Grove, IL, USA). Tissue sections and TM cells incubated without primary antibodies served as a negative control and were used to normalize the fluorescent intensities by background elimination. Sections of non-injected eyes served as a background control for GFP fluorescence. For quantifying staining specific to the mouse TM, a region of interest was drawn around the TM area and represented as the unit of fluorescence intensity per μm^2^. MYOC fluorescent intensity in TM3 cells stably expressing mutant *MYOC* was quantified by imaging thirteen to fifteen different non-overlapping areas of each treated wells. The fluorescent intensity was normalized using number of cells per image as determined by DAPI staining.

### Western blot

TM3 cells were lysed in 1 × RIPA buffer containing protease inhibitors. Cellular lysates were loaded on denaturing 4%–12% gradient polyacrylamide readymade gels (NuPAGE Bis–Tris gels, Life Technologies). The proteins were separated using Invitrogen’s Mini Gel electrophoresis tank at constant voltage (150 V) and transferred onto a methanol-activated PVDF membrane (Immobilon-P, 0.45 μm pore size; Merk Millipore Ltd., St. Louis, MO, USA) as described previously^62^. The blots were blocked with 5% nonfat dry milk prepared in 1 × PBS with Tween-20 (PBST), followed by overnight incubation at 4 °C with respective primary antibodies (1:1000 dilutions). The primary antibodies used were KDEL (catalog# MBP1-97469, Novus Biologicals, Littleton, CO, USA); MYOC (catalog# ab41552, Abcam); ATF4 (catalog# 10835-1-AP, Proteintech); CHOP (catalog# 15204-1-AP, Proteintech; 6003-1395, Novus). GAPDH (catalog# 60004-1-Ig, Proteintech) was used as a loading control. After overnight primary antibody incubation, the blots were washed with 1 × PBST and incubated with respective horseradish-peroxidase (HRP)-conjugated secondary antibodies (1:2500 dilution) and developed with enhanced chemiluminescence (ECL) detection reagent (SuperSignal West Femto Maximum Sensitivity Substrate; Life Technologies). Protein bands were visualized using an LI-COR Biosciences Odyssey-Fc image system (Lincoln, NE, USA) and quantified using ImageStudio software (LI-COR Biosciences) as previously explained^65,66^.

### Genomic endonuclease assay

Genomic DNA was isolated using NucleoSpin Tissue (catalog# 740952, Macherey-Nagel, Allentown, PA, USA) from cells treated with LV_cr*MYOC*, AAV2_cr*MYOC*, LV_Null and AAV2_Null. Untreated cells were used as experimental control. *MYOC*, which is a target of selected gRNA was amplified by PCR. PCR product was denatured and reannealed using the Alt-R Genome Editing Detection Kit protocol (catalog# 1075932, Integrated DNA Technologies, Coralville, Iowa, USA). This generated mismatched heteroduplex DNA products containing strands with CRISPR/Cas9-induced indel reannealed to wild-type strands or different indel. The heteroduplexes were subsequently detected using T7 endonucleases (T7E1), that cleaved the mismatched DNA. The resulting cleaved products were analyzed by gel electrophoresis.

### CRISPR-Cas9 off-target effects by whole genome sequencing (WGS)

TM3 cells were transduced with lentivirus expressing Cas9 only (gScr), or Cas9 with gRNA against myocilin (g*MYOC*). 48 h after infection, genomic DNA was extracted from gScr, g*MYOC*, and parental TM3 (NT) cells. Samples were sequenced on a Novaseq 6000 system at 30 × coverage. The FASTQ files for all three samples (g*MYOC*, gScr, NT) were aligned to the human reference genome (GRCh37) with BWA-mem and sorted with SAMtools^67^. The resulting BAM files were processed to remove duplicate reads with Picard Tools (http://broadinstitute.github.io/picard/). Local realignment and base quality recalibration were performed with Genome Analysis Tollkit (GATK)^68^. The most-likely off-target sites were determined using Cas-OFFinder^69^ based upon the human reference genome (GRCh37), allowing the alignment of the gRNA to the genome to have up to 3 mismatches, DNA bulge size less than or equal to 1, and an RNA bulge size less than or equal to 1. The resulting 1214 unique sites were prioritized using the crisprScore package in Bioconductor, with the CFD algorithm^70^. The top 100 sites were selected based upon their crisprScore. Each site was inspected visually using the Integrated Genome Viewer^71^ with the analysis-ready BAM files for all three samples loaded. Sites were judged to be off target if indels were observed within 20 nt of the target in the g*MYOC* sample and not in any of the other samples. Sites were evaluated for potential transcriptional impact based upon their presence within transcription factor binding sites or transcriptional enhancers. These evaluations used the VISTA Enhancers track (REF)^72^ and the Conserved TFBS track from the UCSC genome browser (https://genome.ucsc.edu).

### Statistics

Statistical analyses were performed using Prism 9.0 software (GraphPad, San Diego, CA, USA). A *P* value of < 0.05 was considered significant. Data was represented as mean ± SEM. An unpaired Student’s *t* test (two-tailed) was used for comparing data with two-groups. The IOP results that comprise more than two groups were analyzed by repeated-measures two-way ANOVA followed by a Bonferroni post-hoc correction.

## Results

### Ocular transduction patterns of various AAV2 serotypes and lentiviral particles in mouse eyes

**Figure 1:**
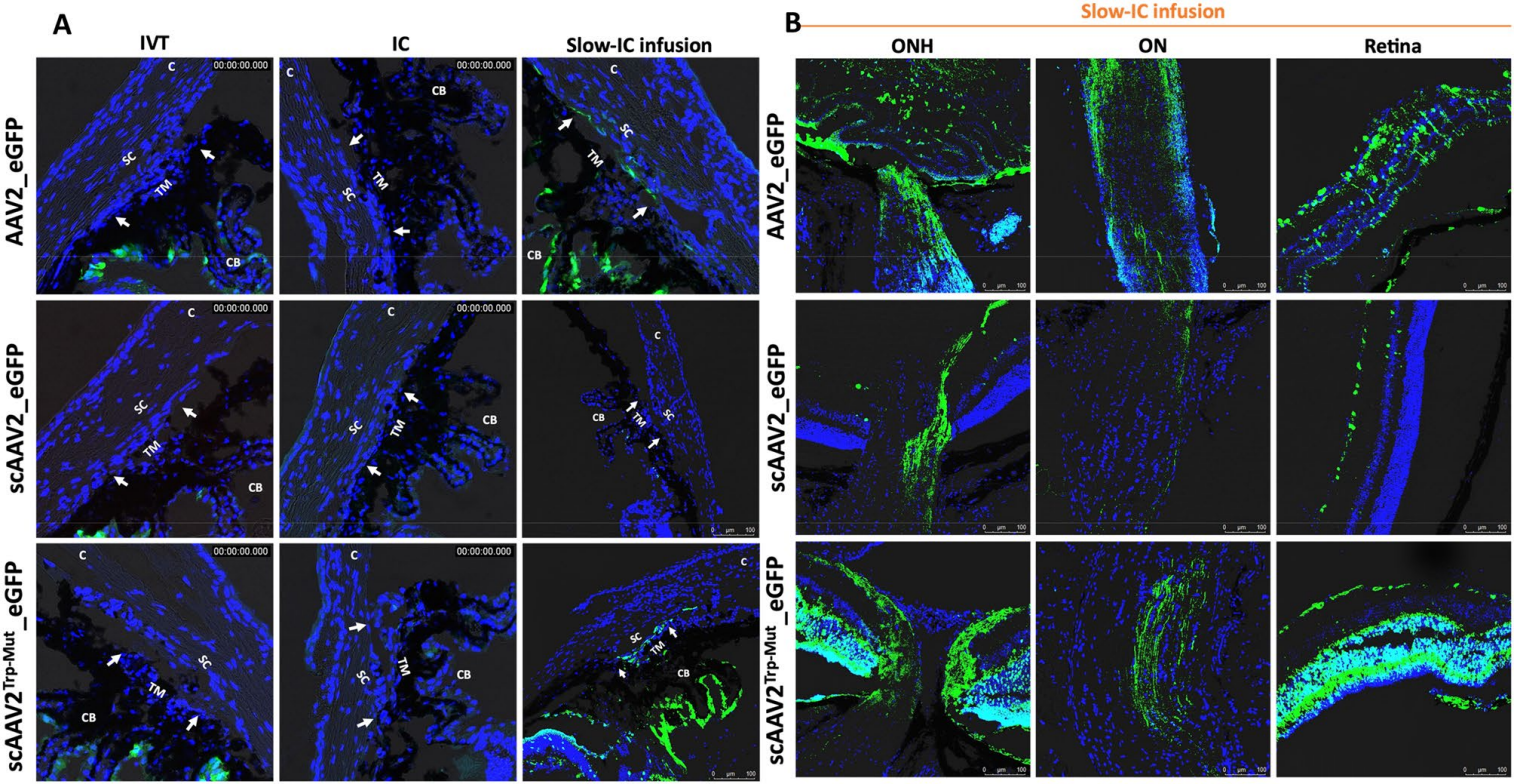
AAV2-mediated GFP transduction in ocular tissues of C57BL/6J mice. ssAAV2, scAAV2 and scAAV2^Trp-Mut^ expressing GFP (2 × 10^10^ GC/eye) were injected in mouse eyes via IVT or IC bolus injections or slow IC infusion (n = 3 eyes each). GFP expression was examined by confocal imaging 2 weeks post-injections in anterior segment (**A**) and retina (**B**). Non-injected eyes serve as control for background fluorescent intensity. TM—trabecular meshwork; SC—Schlemm’s canal; CB—ciliary body; C—cornea; I—iris. White arrows show TM.

**Figure 2:**
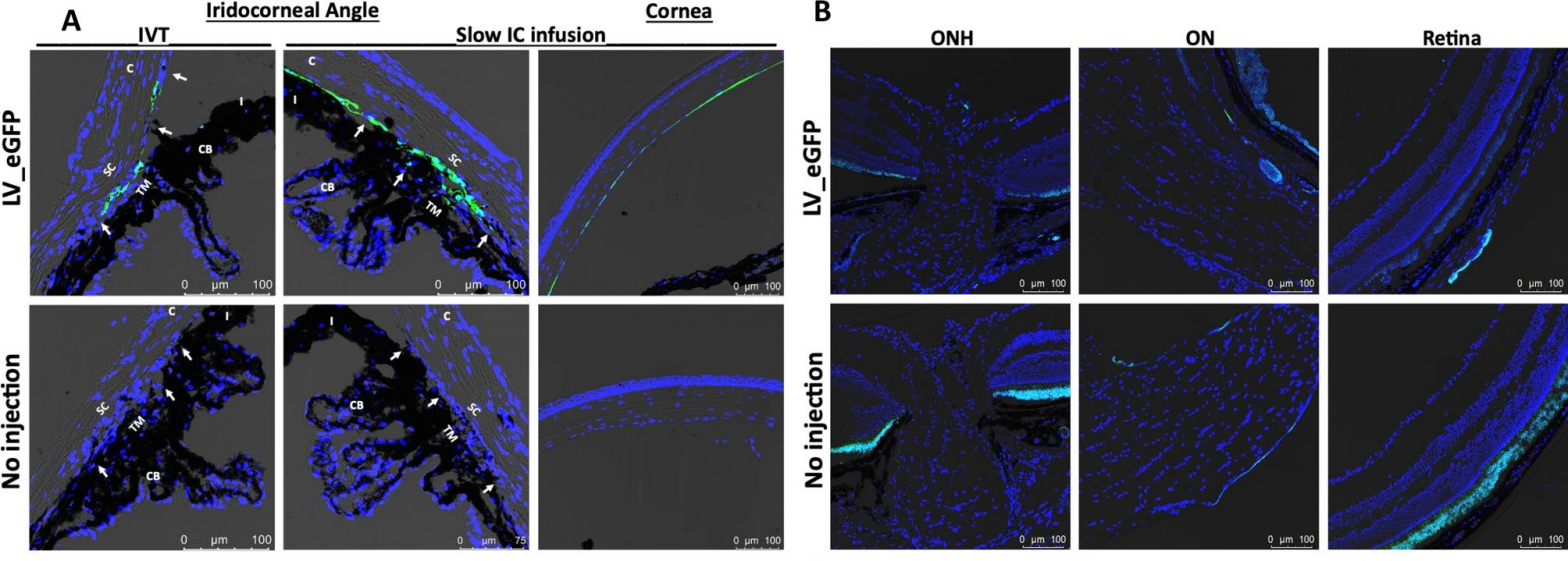
LV-mediated GFP transduction in ocular tissues of C57BL/6J mice. LV particles expressing GFP (2.5 × 10^6^ TU/eyes) were injected in mouse eyes via IVT or IC bolus injections or slow IC infusion (n = 3 eyes each). GFP expression was examined by confocal imaging 2 weeks post-injections in the anterior segment for IVT and IC slow infusion (**A**) and in retina for IVT injections (**B**). Non-injected eyes served as control for background fluorescent intensity. TM—trabecular meshwork; SC—Schlemm’s canal; CB—ciliary body; C—cornea; I—iris. White arrows show TM. Note that variable autofluorescence in RPE region was observed in both control and LV_eGFP injected eyes (**B**).

Selective AAV serotypes were shown to have tropism towards the TM of mice, rats, and monkeys in previous studies^73–75^. These studies suggest that single-stranded (ss)^75^ and self-complimentary (sc) AAV2 efficiently transduces TM^73,76^. However, AAV2 also exhibits strong tropism to other ocular tissues. Our recent study also showed robust tropism of LV to the mouse TM^62^. We therefore compared the TM specific tropism of various AAV2 capsid variants and LV particles expressing GFP. To select which AAV serotypes has the best tropism towards TM, we first screened several AAV2 serotypes in primary human TM cells (SI I). Human primary TM cells (n = 2 strains) were grown to confluency in 12-well plates and treated with AAV2/2, AAV2/4, AAV2/5 and AAV2/8 at multiplicities of infection (MOI) of 2.5 × 10^1^ to 2.5 × 10^3^ viral genomes (VG)/cell as described previously^75^. GFP expression was examined by fluorescent microscopy after 72 h of transduction (SI I). No AAVs caused GFP expression at MOI of 2.5 × 10^1^ VG/cell (not shown), but we observed robust AAV2-GFP expression at MOI of 2.5 × 10^3^ VG/cell. Note that these high MOI are consistent with other cell types^73,75,77^. Based on these data, we chose to further investigate whether various AAV2 capsids produce robust tropism in mouse TM. These viruses were injected via IVT or IC (bolus or slow perfusion) routes to determine GFP expression in ocular tissues (n = 3 for each vector per route of injections) and GFP was examined by confocal imaging 2-weeks post-injection. We chose to perform slow perfusion IC injection because bolus IC injections may wash out quickly through the outflow pathway, which can limit the viruses’ ability to transduce TM cells. None of the three capsid variants, ssAAV2_*GFP*, scAAV2_*GFP* and scAAV2^Trp-Mut^_*GFP* showed GFP expression in the TM region delivered by bolus IVT or IC injections (Fig. 1A). Consistent with previous studies, we observed a robust GFP expression in the retina (data not shown). Slow IC infusion of scAAV2^Trp-Mut^ induced robust GFP expression in TM and other tissues at the iridocorneal angle compared to the ssAAV2 vector (Fig. 1A). However, no GFP fluorescence was observed in the TM of scAAV2 slow IC-infused eyes. Irrespective of their differences in TM transduction efficiency, these AAV2 variants were also found to transduce retina, optic nerve, and optic nerve head regions robustly in slow IC-treated eyes (Fig. 1B). Since AAVs did not show selective and robust tropism to TM, we next evaluated the selective tropism of LV. Consistent with our previous study^62^, IVT bolus injections of LV_*GFP* induced GFP expression in mouse TM (Fig. 2A). This GFP fluorescence was seen throughout the TM. Minor expression was also observed in ciliary body region. Importantly, no GFP expression was observed in the retina confirming the specificity of our LV to the TM (Fig. 2B). For comparative purposes, the efficiency of LV vectors was also examined via slow IC infusion. We observed robust and more efficient TM transduction via the slow-infused IC route (Fig. 2A,B). However, significant LV_*GFP* transduction was also observed in the inner corneal endothelium layer (Fig. 2B). Since IVT delivery of LV demonstrated most selective and efficient tropism to TM, we utilized this approach to deliver *Cas9* to TM in our subsequent studies.

### Comparison of AAV2 and LV-mediated MYOC editing in vitro

**Figure 3:**
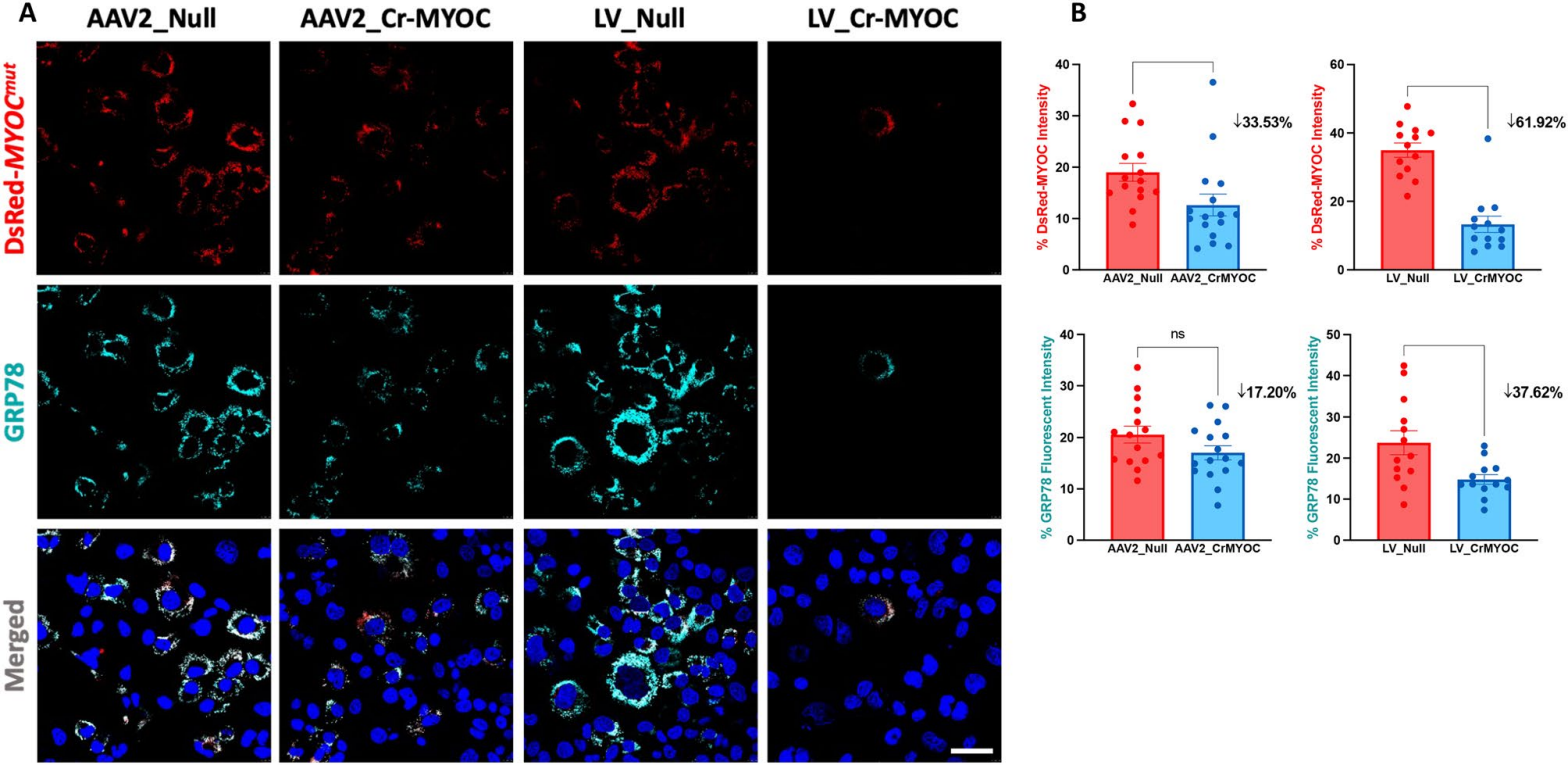
Comparison of AAV2- and LV-mediated *MYOC* editing in TM cells. **(A)** Representative images showing AAV2_cr*MYOC* or LV_cr*MYOC* treatment of TM3 cells stably expressing DsRed tagged mutant *MYOC.* AAV2_cr*MYOC* or LV_cr*MYOC* reduces intracellular *MYOC* and ER stress marker GRP78 (cyan) (scale bar = 50 μm; n = 3). **(B)** Quantitative analysis of fluorescent intensities demonstrates a significant reduction of MYOC fluorescence in both LV and AAV2-cr*MYOC* treated TM cells. For GRP78 immunostaining, only LV_cr*MYOC* cells showed significant decrease. Unpaired (two-tailed) student *t* test, with *p < 0.05, **p < 0.01, ***p < 0.001 and ****p < 0.0001. Quantitative data represented as mean ± SEM.

We have previously demonstrated CRISPR/Cas9-mediated knockout of the *MYOC* gene using the Ad5 delivery system^51^. Here, we examined whether AAV2 and LV expressing cr*MYOC* efficiently edit the *MYOC* gene in human TM3 cells. TM3 cells stably expressing DsRed tagged human *MYOC* with Y437H or G364V mutations (DsRed-*MYOC*^*Mut*^) exhibit reduced secretion and intracellular accumulation of mutant-*MYOC*^35,44,46,51,59,65^. Overall, a decrease in DsRed puncta was observed in TM3 cells transduced with AAV2_cr*MYOC* and LV_cr*MYOC* compared to TM3 cells transduced with controls viral particles (Fig. 3A). We next quantified MYOC accumulation using Image J, which revealed that LV-cr*MYOC* reduced MYOC significantly by 62% while AAV2_cr*MYOC*-mediated reduction was 34% (Fig. 3B). The decrease in MYOC accumulation in LV_cr*MYOC* treated cells was also reflected on GRP78 fluorescence, reduced significantly by 38% compared to the control cells.

**Figure 4:**
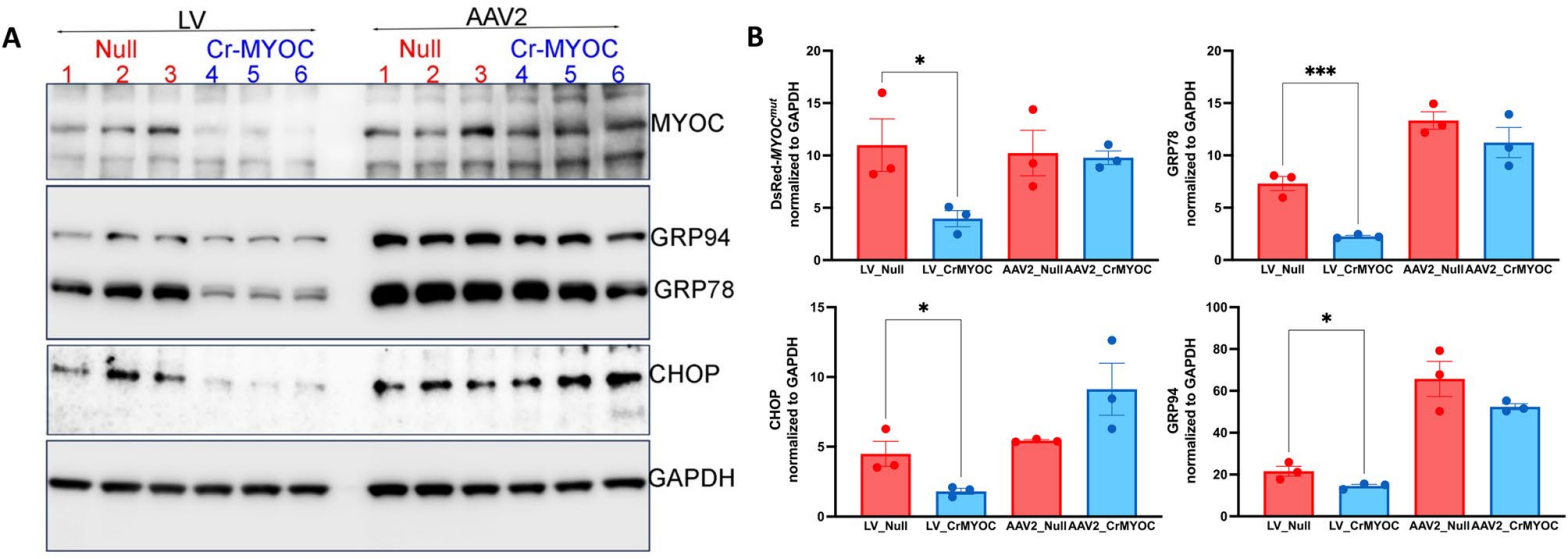
Effect of AAV2- and LV-mediated *MYOC* editing on ER stress markers in TM cells. (**A)** Representative Western blot showing decreased protein levels of MYOC, GRP78, GRP94, and CHOP predominantly in LV_cr*MYOC* treated cells compared to AAV2_cr*MYOC* (n = 3). (**B)** The densitometric analysis confirms significant decrease in MYOC and associated ER stress markers with LV_cr*MYOC* treatment only, with no significant effect observed in AAV2_cr*MYOC* treated cells. Unpaired (two-tailed) student *t* test, with *p < 0.05, **p < 0.01, ***p < 0.001 and ****p < 0.0001. Quantitative data represented as mean ± SEM.

We further determined genome editing efficiency of AAV_cr*MYOC* or LV_cr*MYOC* in TM3 cells stably expressing mutant MYOC using Western blot analysis (Fig. 4A,B). Western blot and its densitometric analysis demonstrated significant reduction in MYOC and ER stress markers (GRP78, CHOP and GRP94) in LV_cr*MYOC*-treated cells compared to cells transduced by LV-null. Although AAV2-cr*MYOC* reduced MYOC and ER stress markers, this reduction was not statistically significant compared to cells treated with AAV2-null. Using the Alt-R Genome Editing Detection Kit, we further confirmed T7 endonuclease (T7E1) induced cleaved product in both our LV_cr*MYOC* and AAV2_cr*MYOC* treated DNA samples (SI II). No cleaved product was observed in untreated control, LV_Null and AAV2_Null treated DNA samples. These data indicate that LV_cr*MYOC* edits *MYOC* and reduces its intracellular accumulation, relieving ER stress in human TM cells.

### LV_cr*MYOC* decreases mutant myocilin in TM and reduces elevated IOP in mouse model of MYOC-associated glaucoma

**Figure 5:**
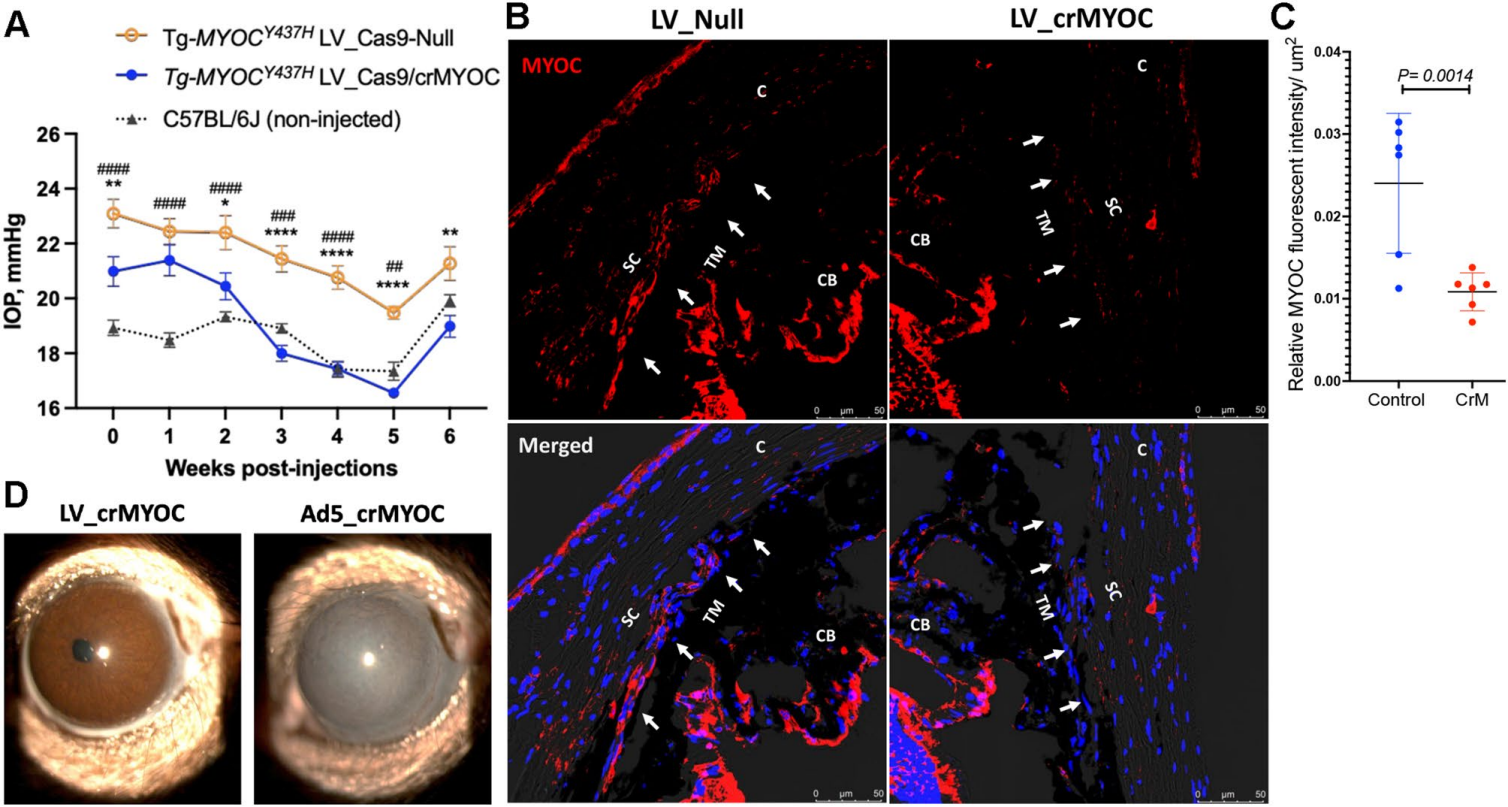
LV-cr*MYOC* knockout of human *MYOC* reduces mutant myocilin in TM and lowers elevated IOP in *Tg-MYOC*^*Y437H*^ mice. **(A)** IOP measurements in *Tg-MYOC*^*Y437H*^ and age-matched C57BL/6J mice. Ocular hypertensive *Tg- MYOC*^*Y437H*^ mice were injected with LV_*Cas9*-Null or LV_cr*MYOC* (2.5 × 10^6^ TU/eyes) and IOPs were measured weekly (n = 6 mice each; > 9 months old). LV_cr*MYOC* reduced elevated IOP significantly compared to ocular hypertensive LV_Null injected *Tg-MYOC*^*Y437H*^ mice. Data represented as mean ± SEM; *p < 0.05, **p < 0.01, ***p < 0.001 and ****p < 0.0001. # represents significant IOP comparison between LV_Null-injected Tg-MYOC^*Y437H*^ mice and aged matched C57BL/6J mice. Two-way ANOVA with repeated measurements and Bonferroni post-hoc analysis were performed. **(B)** Representative images showing decreased MYOC in TM of *Tg-MYOC*^*Y437H*^ mice transduced with LV_cr*MYOC* (n = 6). (**C**) Relative MYOC fluorescence intensity/µm^2^ of the TM region showing significant reduction of MYOC protein in LV_cr*MYOC* injected *Tg- MYOC*^*Y437H*^ mice compared to control mice; n = 6 eyes. Unpaired student *t*-test. (**D**) Representative slit-lamp images revealed no ocular inflammation in *Tg-MYOC*^*Y437H*^ mice injected intravitreally with LV_cr*MYOC* (2.5 × 10^6^ TU/eyes) compared to eyes transduced with Ad5_cr*MYOC* (2 × 10^6^ pfu/eyes; n = 3 each).

Since LV_cr*MYOC* disrupts *MYOC* efficiently in vitro, we further determined whether LV_cr*MYOC* rescues *Tg-MYOC*^*Y437H*^ mice, which expresses human *MYOC* with the Y437H mutation. As shown in Fig. 1, intravitreal injection of LV targets mouse TM. We therefore performed a single intravitreal injection of LV_cr*MYOC* in adult ocular hypertensive *Tg-MYOC*^*Y437H*^ mice. Before injections (0 day), we observed that *Tg-MYOC*^*Y437H*^ mice show IOP elevation compared to WT mice (Fig. 5A). Ocular hypertensive *Tg-MYOC*^*Y437H*^ mice were injected intravitreally with LV_cr*MYOC* or LV_*Cas9*-Null (2.5 × 10^6^ TU/eyes). While LV_Null treated *Tg-MYOC*^*Y437H*^ mice exhibited significant IOP elevation compared to age-matched C57BL/6 J mice, LV_cr*MYOC* mice demonstrated a significant reduction of IOP 3-weeks after injection and IOPs in LV_cr*MYOC* injected mice were similar to WT mice 3-weeks after injection (Fig. 5A). The mean dark-adapted IOP was ~ 17.75 mmHg in LV_cr*MYOC*-injected *Tg-MYOC*^*Y437H*^ mice compared to ~ 21.07 mmHg in LV_Null treated *Tg-MYOC*^*Y437H*^ mice and ~ 18.4 mmHg in control WT mice. We observed that IOP in all three groups is elevated slightly at 6-weeks after injections. Since IOP is relatively higher in all three groups at 6 weeks compared to other time periods, it is likely due to a change in local environment in our facility. We next determined whether LV_cr*MYOC* reduced mutant myocilin accumulation in *Tg-MYOC*^*Y437H*^ mice by immunolabeling of fixed anterior segments with MYOC antibody (Fig. 5B). Immunostaining data revealed that LV_cr*MYOC* treatment reduced MYOC labeling in the TM region of *Tg-MYOC*^*Y437H*^ mice compared to LV_Null treated *Tg-MYOC*^*Y437H*^ mice. We next measured relative MYOC fluorescence intensity in the TM region and its analysis demonstrated significantly reduced MYOC accumulation in LV_cr*MYOC *treated* Tg-MYOC*^*Y437H*^ mice compared to LV_null treated *Tg-MYOC*^*Y437H*^ mice (Fig. 5C).These data indicate that LV_cr*MYOC* edits the *MYOC* gene and prevents IOP elevation in *Tg-MYOC*^*Y437H*^ mice. Since viral vectors including Ad5 tend to cause ocular inflammation, we next examined ocular structures in LV_cr*MYOC* injected *Tg-MYOC*^*Y437H*^ mice compared to Ad5_cr*MYOC* injected *Tg-MYOC*^*Y437H*^ mice using slit lamp imaging and histological analysis of anterior segments (Fig. 5D and SI III). The eyes injected with Ad5_cr*MYOC* (2 × 10^6^ pfu/eyes) developed acute inflammation determined by an opaque white appearance of the anterior segments (Fig. 5D). Moreover, H&E staining of the anterior segment revealed increased corneal thickness in Ad5 injected eyes (SI III). In contrast, both slit lamp imaging and H&E staining revealed that LV_cr*MYOC* injected eyes showed no abnormalities in the anterior segments. These data indicate that an IVT injection of LV_cr*MYOC* causes minimal ocular toxicity in mice.

One of the major concerns with CRISPR-Cas9 based genome editing is off-target effects. To determine the off-target effects due to LV_cr*MYOC*, we performed WGS on TM3 cells transfected with LV_cr*MYOC* or LV_crScrambled. The most obvious change is at the myocilin genome locus (Supplementary information IV and Supplementary information V). Out of the top 100 predicted off-target sites based on crisprScore, two sites including MLLT3 with 27% (7/26) and FAM19A5 with 4% (1/24) were identified as potential changes in TM3 cells treated with LV_cr*MYOC*, but not in cells treated with LV_crScrambled gRNA or parental TM3 (NT) samples. These sites had crisprScores of 0.75 and 0.50 respectively. Both observed changes are in deep intronic regions, located more than 10,000 nt from the nearest exon. A total of nine off-target sites for LV_cr*MYOC* were considered most likely (crisprScore ≥ 0.5), of which only one falls in a coding region. That site is within SLC2A10 and has a crisprScore of 0.53 requiring 3 mismatches and a bulge. We detected the change at the MLLT3 site by the T7 endonuclease 1 assay (T7E1). We cannot detect the change at the FAM19A5 site by the T7E1 assay. This may be due to the lack of sensitivity of the T7E1 assay or sequencing error. The top 109 sites (those with a crisprScore > 0.15) were evaluated for possible regulatory effects. This evaluation identified two weakly-predicted sites, which overlapped potential transcription factor binding sites, an intronic site in LRMDA (72^nd^ highest predicted site; CFD = 0.1888) and one intergenic site (97th highest predicted site; CFD = 0.1599). No overlap with the set of VISTA enhancers was observed. Together, these data indicate that LV_CrMYOC edits *MYOC* with high efficiency with limited off-target effects and little-to-no predicted impact of those off-target effects.

## Discussion

Recent advances in genome editing technologies allow investigators to directly alter the genes associated with disease pathology. The gain-of-function mutation of the *MYOC* gene serves as a direct target for gene editing without the need for gene replacement. Knocking down *MYOC* expression in the eye does not compromise any normal ocular physiological function and it is relatively easy to knock out the gene compared to correcting its mutations^25,39,40^. The eye is a favorable target to develop gene therapy attributed to its ease of accessibility for routine clinic-based applications and the fact that it is an isolated immune privileged compartment separated by the blood-retinal barrier^54,56^. Importantly, long duration of efficacy can be obtained from a single dose of gene delivery, thus eliminating the requirement for patient compliance with routine eye drop application^78^. We have previously demonstrated that Ad5_cr*MYOC* decreases mutant MYOC in TM and rescues glaucoma in transgenic mice. Although Ad5 was used experimentally due to its tropism for the TM, Ad5 is not a suitable viral vector for clinical use due to its immunogenic response^56^. Here, we show that lentiviral particles mediate optimum and efficient *MYOC* editing in TM and prevent IOP elevation in a mouse model of *MYOC*-associated POAG. For clinical application, the selectivity to transduce and target transgene expression in a specific cell region is important to avoid off-site gene editing^78^. The modifications of viral serotypes or capsid can alter the cellular tropism of the viral vector^73,74,79,80^. In addition, the route of vector delivery, the intraocular environment and proximity of the target tissue to the delivery site help determine the efficiency and selectivity of the transduction^80,81^. Based on the anatomy, the IC route provides the most efficient TM transduction in several studies using AAV or LV vectors^54,82^. Most of the anterior segment aqueous humor flow exits via the TM, which is known for its phagocytic property^83^. This further promotes the viral vectors to have high affinity for TM transduction compared to cornea, lens or ciliary body. However, IC bolus injection may force rapid washout of the viral particles, with limited exposure to the target tissue, especially in the mouse which has a very small eye. Hence, we employed slow-IC infusion that delivers the virus for an extended period. In contrast, the IVT injection route provides a longer-lasting depot effect for sustained release of the injected vectors, proving to be an efficient route for gene therapy application with single dose administration. Our findings indicate that slow-IC infusion is the most efficient route for inducing robust transgene expression in the TM via both AAV2 capsid variants and LV vectors. However, LV vectors induced GFP expression in the corneal endothelium, which is consistent with a previous study^84^. Nonetheless, the IVT route for LV_*GFP* proved to be more specific and selective in transducing the mouse TM, with minor GFP expression noted in the ciliary body region. The slow and smaller release of the virus particles from the posterior vitreous, prevent their proximity and exposure to corneal endothelial, enough to reduce the propensity to transduce.

The AAV vectors are well known for their safety and efficacy in clinical application and are the preferred option for retinal gene therapy^56,78^. This nonpathogenic ssDNA and replication deficient parvovirus provides long-term transgene expression, with only a mild immunogenic response. However, they are limited by their ability to transduce the tissues of the anterior segments. Several studies have emphasized the use of scAAV capsids or their mutant forms for efficient transduction of TM cells as they facilitate the generation of dsDNA^73,74,85^. However, the size of the transgene cassette that can be inserted is very limited. In contrast, the capsid mutation of AAV serotype 2 (AAV2) have better TM transduction via the intracameral route in rodents, perfused anterior chamber, and cultured human TM cells, thus resolving the issue associated with transgene insertion size^75^. While evaluating cellular tropism of AAV2 serotype capsid variants via GFP expression, the scAAV2^Trp-Mut^ induced prominent expression of GFP in TM via the slow-IC infusion route. TM transduction was also observed with our ssAAV2 capsid variant. However, we demonstrate that AAV2 is not selective to TM, with robust GFP expression observed in retina and ONH. The selectivity of transgene expression can also be determined by use of tissue specific promoters. The CMV promoters used in our vector constructs promotes ubiquitous transgene expression in a majority of ocular tissues including corneal endothelium, non-pigmentary epithelial cells and retinal tissues^86^. A few studies have reported TM preferential promoters such as matrix Gla protein and chitinase-3-like-1 promoter^80,87^. This non-specificity of AAVs to ocular tissues can increase Cas9-associated off-target effects, thus limiting its clinical applications for the treatment of glaucoma.

Lentiviruses are known for their capacity to induce sustained transgene expression with low immunogenic response. Both FIV and HIV based LV are used in ocular research^82^. LV vector efficiency is currently being investigated in two macular degeneration clinical trials^78,81^. Our HIV based VSV-G pseudotyped vector proved to be selective towards the mouse TM via the IVT route. The ssRNA genome of lentivirus is reverse transcribed into dsDNA that becomes integrated into the host genome via integrase enzyme activity. This is one of the major limitations of using LV in clinical applications. Based on the recent advancement, our LV vectors are designed to avert insertional mutagenesis by inhibiting integrase. These integrase-deficient lentivirus vectors can be generated by introducing non-pleiotropic mutations within the open reading frame that specifically targets the integration function without affecting the life cycle of the virus^88^.

LVs are known for their high transgene loading capacity (7 kb), which is a major advantage over the AAV vectors (~ 4.6 kb). Therefore, they are more suitable for packaging gene editing constructs such as CRISPR/Cas9. Although scAAV vectors have higher TM transduction efficiency as reported by a previous study^85^, they are limited by the capacity for packaging the cargo gene. Therefore, we used ssAAV2 variants to determine the efficiency of CRISPR/Cas9 based *MYOC* gene editing. Moreover, we used SaCas9 for ssAAV2 based CRISPR assembly, as it is smaller in size compared to the SpCas9^89–91^. Both LV and AAV2 expressing Cas9 were able to edit the *MYOC* gene in human TM cells. However, the overall effect of *MYOC* gene editing on MYOC protein levels and ER stress was more significantly pronounced in LV-treated cells compared to AAV2-treated cells. Comparable to our previous study^51^, our LV_cr*MYOC* was able to knock down *MYOC* expression in transduced TM of *Tg-MYOC*^*Y437*^^*H*^ mice, resulting in significantly reduced IOP independent of any immunogenic response. We and others have shown that Ad5 carrying genetic material induces ocular inflammation compared to Ad5 carrying an empty cassette^64,92^, which is a major limitation for its clinical applications. Consistent with this, we observed that Ad5_crMYOC induced ocular inflammation while LV_crMYOC did not show any signs of ocular inflammation further supporting its clinical safety.

One serious concern with traditional Cas9 is off-target effects, which occur due to non-selectivity of Cas9 to similar genomic regions. Traditional nuclease CRISPR/Cas9-based gene knockouts also introduce DNA double-strand breaks (DSBs), which pose serious risks such as large deletions, translocations, and chromosomal abnormalities. In addition, this effect can be more pronounced when Cas9 is expressed for longer period as in the case when delivered using viral vectors. WGS revealed that our LV_cr*MYOC* targets *MYOC* in TM cells with high efficiency but we have also observed limited off-target effects in LV_cr*MYOC* treated TM cells. We utilized in silico tools to select our gRNA targeting *MYOC*. These in silico tools search for potential off-target sites in the whole genome and calculate the likelihood of off-target editing. Most off-target effects are often gRNA dependent and selecting another gRNA may reduce these off-target effects. In addition,viral vectors tend to cause prolonged expression of Cas9, which can increase off-target effects^93^. To overcome these concerns, our future studies will be directed towards utilizing base editors and non-viral delivery approaches. Recent advances made in precision genome editing offers better promise in reducing these off-target effects^94–96^. Specially, adenine base editors, comprise a catalytically impaired Cas9 (nCas9) with adenosine deaminase (TadA) and enable the conversion of A•T to G•C with high precision and efficiency without causing DNA double strand breaks^97^. Base editors may exhibit some bystander effect in nearby regions with little or no off-target effects. Since we are knocking out *MYOC*, this may not cause any serious issues. Our future experiments will be directed towards adapting precision genome editing for glaucoma. Several studies have recently utilized non-viral delivery platforms such as lipid nanoparticles to deliver Cas9 mRNA or protein for optimum gene editing with minimum off-target effects^96,98–100^. These non-viral deliveries of base editors provide a promising lead for efficient gene editing in ocular diseases with minimum off-target effects.

In conclusion, our studies show that LVs are highly efficient in delivering Cas9 to TM without any ocular toxicity and LV-mediated gene editing is highly efficient in reducing mutant myocilin and lowering elevated IOP in mouse model of glaucoma. Importantly, our studies lay the foundation for further development of gene editing methods effectively treat MYOC-associated glaucoma.

### Supplementary Information

Supplementary Information 1.

Supplementary Information 2.

Raw data.

## Data Availability

All the datasets used and/or analysed in the present study is available from the corresponding author on reasonable request.

## References

[1] Quigley HA, Broman AT (2006). The number of people with glaucoma worldwide in 2010 and 2020. Br. J. Ophthalmol..

[2] Thylefors B, Negrel AD (1994). The global impact of glaucoma. Bull. World Health Organ..

[3] Tham YC, Li X, Wong TY, Quigley HA, Aung T, Cheng CY (2014). Global prevalence of glaucoma and projections of glaucoma burden through 2040: A systematic review and meta-analysis. Ophthalmology..

[4] Nickells RW (1999). Apoptosis of retinal ganglion cells in glaucoma: An update of the molecular pathways involved in cell death. Surv. Ophthalmol..

[5] Quigley HA (1999). Neuronal death in glaucoma. Prog. Retin. Eye Res..

[6] Davis BM, Crawley L, Pahlitzsch M, Javaid F, Cordeiro MF (2016). Glaucoma: The retina and beyond. Acta Neuropathol..

[7] Kwon YH, Fingert JH, Kuehn MH, Alward WL (2009). Primary open-angle glaucoma. N. Engl. J. Med..

[8] Rohen JW (1983). Why is intraocular pressure elevated in chronic simple glaucoma? Anatomical considerations. Ophthalmology..

[9] Weinreb RN, Aung T, Medeiros FA (2014). The pathophysiology and treatment of glaucoma: A review. JAMA..

[10] Distelhorst JS, Hughes GM (2003). Open-angle glaucoma. Am. Fam. Phys..

[11] Braunger BM, Fuchshofer R, Tamm ER (2015). The aqueous humor outflow pathways in glaucoma: A unifying concept of disease mechanisms and causative treatment. Eur. J. Pharm. Biopharm..

[12] Iglesias AI, Springelkamp H, Ramdas WD, Klaver CC, Willemsen R, van Duijn CM (2015). Genes, pathways, and animal models in primary open-angle glaucoma. Eye (Lond)..

[13] Liu Y, Allingham RR (2017). Major review: Molecular genetics of primary open-angle glaucoma. Exp. Eye Res..

[14] Alward WL (2000). The genetics of open-angle glaucoma: The story of GLC1A and myocilin. Eye (Lond)..

[15] Jonas JB, Aung T, Bourne RR, Bron AM, Ritch R, Panda-Jonas S (2017). Glaucoma. Lancet..

[16] Stone EM, Fingert JH, Alward WL, Nguyen TD, Polansky JR, Sunden SL, Nishimura D, Clark AF, Nystuen A, Nichols BE, Mackey DA, Ritch R, Kalenak JW, Craven ER, Sheffield VC (1997). Identification of a gene that causes primary open angle glaucoma. Science..

[17] Sheffield VC, Stone EM, Alward WL, Drack AV, Johnson AT, Streb LM, Nichols BE (1993). Genetic linkage of familial open angle glaucoma to chromosome 1q21-q31. Nat. Genet..

[18] Ortego J, Escribano J, Coca-Prados M (1997). Cloning and characterization of subtracted cDNAs from a human ciliary body library encoding TIGR, a protein involved in juvenile open angle glaucoma with homology to myosin and olfactomedin. FEBS Lett..

[19] Johnson DH (2000). Myocilin and glaucoma: A TIGR by the tail?. Arch. Ophthalmol..

[20] Shimizu S, Lichter PR, Johnson AT, Zhou Z, Higashi M, Gottfredsdottir M, Othman M, Moroi SE, Rozsa FW, Schertzer RM, Clarke MS, Schwartz AL, Downs CA, Vollrath D, Richards JE (2000). Age-dependent prevalence of mutations at the GLC1A locus in primary open-angle glaucoma. Am. J. Ophthalmol..

[21] Fingert JH, Heon E, Liebmann JM, Yamamoto T, Craig JE, Rait J, Kawase K, Hoh ST, Buys YM, Dickinson J, Hockey RR, Williams-Lyn D, Trope G, Kitazawa Y, Ritch R, Mackey DA, Alward WL, Sheffield VC, Stone EM (1999). Analysis of myocilin mutations in 1703 glaucoma patients from five different populations. Hum. Mol. Genet..

[22] Tamm ER (2002). Myocilin and glaucoma: Facts and ideas. Prog. Retin. Eye Res..

[23] Lohano MK, Su L, Ruochen W, Basnet BB, Naveed H, Khemani VD, Khooharo AA (2016). Myocilin and glaucoma its risk factors. Int. J. Pure Appl. Biosci..

[24] Donegan RK, Lieberman RL (2016). Discovery of molecular therapeutics for glaucoma: Challenges, successes, and promising directions. J. Med. Chem..

[25] Sharma R, Grover A (2021). Myocilin-associated Glaucoma: A historical perspective and recent research progress. Mol. Vis..

[26] Karali A, Russell P, Stefani FH, Tamm ER (2000). Localization of myocilin/trabecular meshwork–inducible glucocorticoid response protein in the human eye. Invest. Ophthalmol. Vis. Sci..

[27] Kubota R, Noda S, Wang Y, Minoshima S, Asakawa S, Kudoh J, Mashima Y, Oguchi Y, Shimizu N (1997). A novel myosin-like protein (myocilin) expressed in the connecting cilium of the photoreceptor: Molecular cloning, tissue expression, and chromosomal mapping. Genomics..

[28] Takahashi H, Noda S, Imamura Y, Nagasawa A, Kubota R, Mashima Y, Kudoh J, Oguchi Y, Shimizu N (1998). Mouse myocilin (Myoc) gene expression in ocular tissues. Biochem. Biophys. Res. Commun..

[29] Fingert JH, Ying L, Swiderski RE, Nystuen AM, Arbour NC, Alward WL, Sheffield VC, Stone EM (1998). Characterization and comparison of the human and mouse GLC1A glaucoma genes. Genome Res..

[30] Wang H, Li M, Zhang Z, Xue H, Chen X, Ji Y (2019). Physiological function of myocilin and its role in the pathogenesis of glaucoma in the trabecular meshwork (Review). Int. J. Mol. Med..

[31] Fingert JH, Stone EM, Sheffield VC, Alward WL (2002). Myocilin glaucoma. Surv. Ophthalmol..

[32] Resch ZT, Fautsch MP (2009). Glaucoma-associated myocilin: A better understanding but much more to learn. Exp. Eye Res..

[33] Aroca-Aguilar JD, Sanchez-Sanchez F, Ghosh S, Fernandez-Navarro A, Coca-Prados M, Escribano J (2011). Interaction of recombinant myocilin with the matricellular protein SPARC: Functional implications. Invest. Ophthalmol. Vis. Sci..

[34] Atienzar-Aroca R, Ferre-Fernandez JJ, Tevar A, Bonet-Fernandez JM, Cabanero MJ, Ruiz-Pastor MJ, Cuenca N, Aroca-Aguilar JD, Escribano J (2022). Transgenic overexpression of myocilin leads to variable ocular anterior segment and retinal alterations associated with extracellular matrix abnormalities in Adult Zebrafish. Int. J. Mol. Sci..

[35] Jacobson N, Andrews M, Shepard AR, Nishimura D, Searby C, Fingert JH, Hageman G, Mullins R, Davidson BL, Kwon YH, Alward WL, Stone EM, Clark AF, Sheffield VC (2001). Non-secretion of mutant proteins of the glaucoma gene myocilin in cultured trabecular meshwork cells and in aqueous humor. Hum. Mol. Genet..

[36] Liu Y, Vollrath D (2004). Reversal of mutant myocilin non-secretion and cell killing: Implications for glaucoma. Hum. Mol. Genet..

[37] Aroca-Aguilar JD, Sanchez-Sanchez F, Ghosh S, Coca-Prados M, Escribano J (2005). Myocilin mutations causing glaucoma inhibit the intracellular endoproteolytic cleavage of myocilin between amino acids Arg226 and Ile227. J. Biol. Chem..

[38] Caballero M, Borras T (2001). Inefficient processing of an olfactomedin-deficient myocilin mutant: Potential physiological relevance to glaucoma. Biochem. Biophys. Res. Commun..

[39] Gould DB, Miceli-Libby L, Savinova OV, Torrado M, Tomarev SI, Smith RS, John SW (2004). Genetically increasing Myoc expression supports a necessary pathologic role of abnormal proteins in glaucoma. Mol. Cell Biol..

[40] Kim BS, Savinova OV, Reedy MV, Martin J, Lun Y, Gan L, Smith RS, Tomarev SI, John SW, Johnson RL (2001). Targeted disruption of the myocilin gene (Myoc) suggests that human glaucoma-causing mutations are gain of function. Mol. Cell Biol..

[41] Gould DB, Reedy M, Wilson LA, Smith RS, Johnson RL, John SW (2006). Mutant myocilin nonsecretion in vivo is not sufficient to cause glaucoma. Mol. Cell Biol..

[42] Wiggs JL, Vollrath D (2001). Molecular and clinical evaluation of a patient hemizygous for TIGR/MYOC. Arch. Ophthalmol..

[43] Shepard AR, Jacobson N, Millar JC, Pang IH, Steely HT, Searby CC, Sheffield VC, Stone EM, Clark AF (2007). Glaucoma-causing myocilin mutants require the Peroxisomal targeting signal-1 receptor (PTS1R) to elevate intraocular pressure. Hum. Mol. Genet..

[44] Joe MK, Sohn S, Hur W, Moon Y, Choi YR, Kee C (2003). Accumulation of mutant myocilins in ER leads to ER stress and potential cytotoxicity in human trabecular meshwork cells. Biochem. Biophys. Res. Commun..

[45] Gobeil S, Letartre L, Raymond V (2006). Functional analysis of the glaucoma-causing TIGR/myocilin protein: Integrity of amino-terminal coiled-coil regions and olfactomedin homology domain is essential for extracellular adhesion and secretion. Exp. Eye Res..

[46] Zode GS, Kuehn MH, Nishimura DY, Searby CC, Mohan K, Grozdanic SD, Bugge K, Anderson MG, Clark AF, Stone EM, Sheffield VC (2011). Reduction of ER stress via a chemical chaperone prevents disease phenotypes in a mouse model of primary open angle glaucoma. J. Clin. Invest..

[47] McDowell CM, Luan T, Zhang Z, Putliwala T, Wordinger RJ, Millar JC, John SW, Pang IH, Clark AF (2012). Mutant human myocilin induces strain specific differences in ocular hypertension and optic nerve damage in mice. Exp Eye Res..

[48] Senatorov V, Malyukova I, Fariss R, Wawrousek EF, Swaminathan S, Sharan SK, Tomarev S (2006). Expression of mutated mouse myocilin induces open-angle glaucoma in transgenic mice. J. Neurosci..

[49] Perez Rojo F, Nyman RKM, Johnson AAT, Navarro MP, Ryan MH, Erskine W, Kaur P (2018). CRISPR-Cas systems: Ushering in the new genome editing era. Bioengineered..

[50] Hsu PD, Lander ES, Zhang F (2014). Development and applications of CRISPR-Cas9 for genome engineering. Cell..

[51] Jain A, Zode G, Kasetti RB, Ran FA, Yan W, Sharma TP, Bugge K, Searby CC, Fingert JH, Zhang F, Clark AF, Sheffield VC (2017). CRISPR-Cas9-based treatment of myocilin-associated glaucoma. Proc. Natl. Acad. Sci. U.S.A..

[52] Shepard AR, Millar JC, Pang IH, Jacobson N, Wang WH, Clark AF (2010). Adenoviral gene transfer of active human transforming growth factor-beta2 elevates intraocular pressure and reduces outflow facility in rodent eyes. Invest. Ophthalmol. Vis. Sci..

[53] Millar JC, Sundaresan Y, Zode GS, Clark AF (2023). Viral vector-induced ocular hypertension in mice. Methods Mol. Biol..

[54] Komaromy AM, Koehl KL, Park SA (2021). Looking into the future: Gene and cell therapies for glaucoma. Vet. Ophthalmol..

[55] Pang IH, Clark AF (2020). Inducible rodent models of glaucoma. Prog. Retin. Eye Res..

[56] Wasnik VB, Thool AR (2022). Ocular gene therapy: A literature review with focus on current clinical trials. Cureus..

[57] Cheng SY, Punzo C (2022). Update on viral gene therapy clinical trials for retinal diseases. Hum. Gene Ther..

[58] Rodriguez-Estevez L, Asokan P, Borras T (2020). Transduction optimization of AAV vectors for human gene therapy of glaucoma and their reversed cell entry characteristics. Gene Ther..

[59] Kasetti RB, Phan TN, Millar JC, Zode GS (2016). Expression of mutant myocilin induces abnormal intracellular accumulation of selected extracellular matrix proteins in the trabecular meshwork. Invest.Ophthalmol. Vis. Sci..

[60] Zode GS, Bugge KE, Mohan K, Grozdanic SD, Peters JC, Koehn DR, Anderson MG, Kardon RH, Stone EM, Sheffield VC (2012). Topical ocular sodium 4-phenylbutyrate rescues glaucoma in a myocilin mouse model of primary open-angle glaucoma. Invest. Ophthalmol. Vis. Sci..

[61] Wordinger RJ, Fleenor DL, Hellberg PE, Pang IH, Tovar TO, Zode GS, Fuller JA, Clark AF (2007). Effects of TGF-beta2, BMP-4, and gremlin in the trabecular meshwork: Implications for glaucoma. Invest. Ophthalmol. Vis. Sci..

[62] Patil SV, Kasetti RB, Millar JC, Zode GS (2022). A novel mouse model of TGFbeta2-induced ocular hypertension using lentiviral gene delivery. Int. J. Mol. Sci..

[63] Wang WH, Millar JC, Pang IH, Wax MB, Clark AF (2005). Noninvasive measurement of rodent intraocular pressure with a rebound tonometer. Invest. Ophthalmol. Vis. Sci..

[64] Kasetti RB, Patel PD, Maddineni P, Patil S, Kiehlbauch C, Millar JC, Searby CC, Raghunathan V, Sheffield VC, Zode GS (2020). ATF4 leads to glaucoma by promoting protein synthesis and ER client protein load. Nat. Commun..

[65] Kasetti RB, Maddineni P, Kiehlbauch C, Patil S, Searby CC, Levine B, Sheffield VC, Zode GS (2021). Autophagy stimulation reduces ocular hypertension in a murine glaucoma model via autophagic degradation of mutant myocilin. JCI Insight..

[66] Zode GS, Sharma AB, Lin X, Searby CC, Bugge K, Kim GH, Clark AF, Sheffield VC (2014). Ocular-specific ER stress reduction rescues glaucoma in murine glucocorticoid-induced glaucoma. J. Clin. Invest..

[67] Li H, Handsaker B, Wysoker A, Fennell T, Ruan J, Homer N, Marth G, Abecasis G, Durbin R, Genome Project Data Processing S (2009). The sequence alignment/map format and SAMtools. Bioinformatics..

[68] McKenna A, Hanna M, Banks E, Sivachenko A, Cibulskis K, Kernytsky A, Garimella K, Altshuler D, Gabriel S, Daly M, DePristo MA (2010). The Genome Analysis Toolkit: A MapReduce framework for analyzing next-generation DNA sequencing data. Genome Res..

[69] Bae S, Park J, Kim JS (2014). Cas-OFFinder: A fast and versatile algorithm that searches for potential off-target sites of Cas9 RNA-guided endonucleases. Bioinformatics..

[70] Hoberecht L, Perampalam P, Lun A, Fortin JP (2022). A comprehensive Bioconductor ecosystem for the design of CRISPR guide RNAs across nucleases and technologies. Nat. Commun..

[71] Robinson JT, Thorvaldsdottir H, Winckler W, Guttman M, Lander ES, Getz G, Mesirov JP (2011). Integrative genomics viewer. Nat. Biotechnol..

[72] Visel A, Minovitsky S, Dubchak I, Pennacchio LA (2007). VISTA Enhancer Browser–a database of tissue-specific human enhancers. Nucl. Acids Res..

[73] Bogner B, Boye SL, Min SH, Peterson JJ, Ruan Q, Zhang Z, Reitsamer HA, Hauswirth WW, Boye SE (2015). Capsid mutated adeno-associated virus delivered to the anterior chamber results in efficient transduction of trabecular meshwork in mouse and rat. PLoS ONE..

[74] Hickey DG, Edwards TL, Barnard AR, Singh MS, de Silva SR, McClements ME, Flannery JG, Hankins MW, MacLaren RE (2017). Tropism of engineered and evolved recombinant AAV serotypes in the rd1 mouse and ex vivo primate retina. Gene Ther..

[75] Wang L, Xiao R, Andres-Mateos E, Vandenberghe LH (2017). Single stranded adeno-associated virus achieves efficient gene transfer to anterior segment in the mouse eye. PLoS ONE..

[76] Borras T, Xue W, Choi VW, Bartlett JS, Li G, Samulski RJ, Chisolm SS (2006). Mechanisms of AAV transduction in glaucoma-associated human trabecular meshwork cells. J. Gene Med..

[77] Ellis BL (2013). A survey of ex vivo/in vitro transduction efficiency of mammalian primary cells and cell lines with Nine natural adeno-associated virus (AAV1–9) and one engineered adeno-associated virus serotype. Virol. J..

[78] Borras T (2017). The pathway from genes to gene therapy in glaucoma: A review of possibilities for using genes as glaucoma drugs. Asia Pac. J. Ophthalmol. (Phila).

[79] Cronin J, Zhang XY, Reiser J (2005). Altering the tropism of lentiviral vectors through pseudotyping. Curr. Gene Ther..

[80] Dang Y, Loewen R, Parikh HA, Roy P, Loewen NA (2017). Gene transfer to the outflow tract. Exp. Eye Res..

[81] Arsenijevic Y, Berger A, Udry F, Kostic C (2022). Lentiviral vectors for ocular gene therapy. Pharmaceutics..

[82] Balaggan KS, Ali RR (2012). Ocular gene delivery using lentiviral vectors. Gene Ther..

[83] Keller KE, Bhattacharya SK, Borras T, Brunner TM, Chansangpetch S, Clark AF, Dismuke WM, Du Y, Elliott MH, Ethier CR, Faralli JA, Freddo TF, Fuchshofer R, Giovingo M, Gong H, Gonzalez P, Huang A, Johnstone MA, Kaufman PL, Kelley MJ, Knepper PA, Kopczynski CC, Kuchtey JG, Kuchtey RW, Kuehn MH, Lieberman RL, Lin SC, Liton P, Liu Y, Lutjen-Drecoll E, Mao W, Masis-Solano M, McDonnell F, McDowell CM, Overby DR, Pattabiraman PP, Raghunathan VK, Rao PV, Rhee DJ, Chowdhury UR, Russell P, Samples JR, Schwartz D, Stubbs EB, Tamm ER, Tan JC, Toris CB, Torrejon KY, Vranka JA, Wirtz MK, Yorio T, Zhang J, Zode GS, Fautsch MP, Peters DM, Acott TS, Stamer WD (2018). Consensus recommendations for trabecular meshwork cell isolation, characterization and culture. Exp. Eye Res..

[84] Challa P, Luna C, Liton PB, Chamblin B, Wakefield J, Ramabhadran R, Epstein DL, Gonzalez P (2005). Lentiviral mediated gene delivery to the anterior chamber of rodent eyes. Mol. Vis..

[85] Buie LK, Rasmussen CA, Porterfield EC, Ramgolam VS, Choi VW, Markovic-Plese S, Samulski RJ, Kaufman PL, Borras T (2010). Self-complementary AAV virus (scAAV) safe and long-term gene transfer in the trabecular meshwork of living rats and monkeys. Invest. Ophthalmol. Vis. Sci..

[86] Everett RS, Evans HK, Hodges BL, Ding EY, Serra DM, Amalfitano A (2004). Strain-specific rate of shutdown of CMV enhancer activity in murine liver confirmed by use of persistent [E1(-), E2b(-)] adenoviral vectors. Virology..

[87] Xiang Y, Li B, Wang JM, Li GG, Zhang H, Manyande A, Tian XB (2014). Gene transfer to human trabecular meshwork cells in vitro and ex vivo using HIV-based lentivirus. Int. J. Ophthalmol..

[88] Rittiner JE, Moncalvo M, Chiba-Falek O, Kantor B (2020). Gene-editing technologies paired with viral vectors for translational research into neurodegenerative diseases. Front. Mol. Neurosci..

[89] Kleinstiver BP, Prew MS, Tsai SQ, Nguyen NT, Topkar VV, Zheng Z, Joung JK (2015). Broadening the targeting range of *Staphylococcus aureus* CRISPR-Cas9 by modifying PAM recognition. Nat. Biotechnol..

[90] Koonin EV, Makarova KS, Zhang F (2017). Diversity, classification and evolution of CRISPR-Cas systems. Curr. Opin. Microbiol..

[91] Hayashi H, Kubo Y, Izumida M, Matsuyama T (2020). Efficient viral delivery of Cas9 into human safe harbor. Sci. Rep..

[92] Shepard AR, Millar JC, Pang IH, Jacobson N, Wang WH, Clark AF (2010). adenoviral gene transfer of active human transforming growth factor-beta 2 elevates intraocular pressure and reduces outflow facility in rodent eyes. Invest. Ophth. Vis. Sci..

[93] O'Geen H, Henry IM, Bhakta MS, Meckler JF, Segal DJ (2015). A genome-wide analysis of Cas9 binding specificity using ChIP-seq and targeted sequence capture. Nucl. Acids Res..

[94] Gaudelli NM, Komor AC, Rees HA, Packer MS, Badran AH, Bryson DI, Liu DR (2017). Programmable base editing of A*T to G*C in genomic DNA without DNA cleavage. Nature..

[95] Banskota S, Raguram A, Suh S, Du SW, Davis JR, Choi EH, Wang X, Nielsen SC, Newby GA, Randolph PB, Osborn MJ, Musunuru K, Palczewski K, Liu DR (2022). Engineered virus-like particles for efficient in vivo delivery of therapeutic proteins. Cell..

[96] Sun D, Sun W, Gao SQ, Lehrer J, Naderi A, Wei C, Lee S, Schilb AL, Scheidt J, Hall RC, Traboulsi EI, Palczewski K, Lu ZR (2022). Effective gene therapy of Stargardt disease with PEG-ECO/pGRK1-ABCA4-S/MAR nanoparticles. Mol. Ther. Nucl. Acids..

[97] Suh S, Choi EH, Raguram A, Liu DR, Palczewski K (2022). Precision genome editing in the eye. Proc. Natl. Acad. Sci. U.S.A..

[98] O'Keeffe Ahern J, Lara-Saez I, Zhou D, Murillas R, Bonafont J, Mencia A, Garcia M, Manzanares D, Lynch J, Foley R, Xu Q, Sigen A, Larcher F, Wang W (2022). Non-viral delivery of CRISPR-Cas9 complexes for targeted gene editing via a polymer delivery system. Gene Ther..

[99] Han JP, Kim M, Choi BS, Lee JH, Lee GS, Jeong M, Lee Y, Kim EA, Oh HK, Go N, Lee H, Lee KJ, Kim UG, Lee JY, Kim S, Chang J, Lee H, Song DW, Yeom SC (2022). In vivo delivery of CRISPR-Cas9 using lipid nanoparticles enables antithrombin gene editing for sustainable hemophilia A and B therapy. Sci. Adv..

[100] Finn JD (2018). A single administration of CRISPR/Cas9 lipid nanoparticles achieves robust and persistent in vivo genome editing. Cell Rep..

