# Supplementary material for: Lentiviral mediated delivery of CRISPR/Cas9 reduces intraocular pressure in a mouse model of myocilin glaucoma
Source: Sci Rep. 2024 Mar 23;14:6958. doi: 10.1038/s41598-024-57286-6 (PMC10960846; doi:10.1038/s41598-024-57286-6)
Supplement: Supplementary file 1 — Supplementary Information 1. [file 41598_2024_57286_MOESM1_ESM.pptx]

## Slide 1
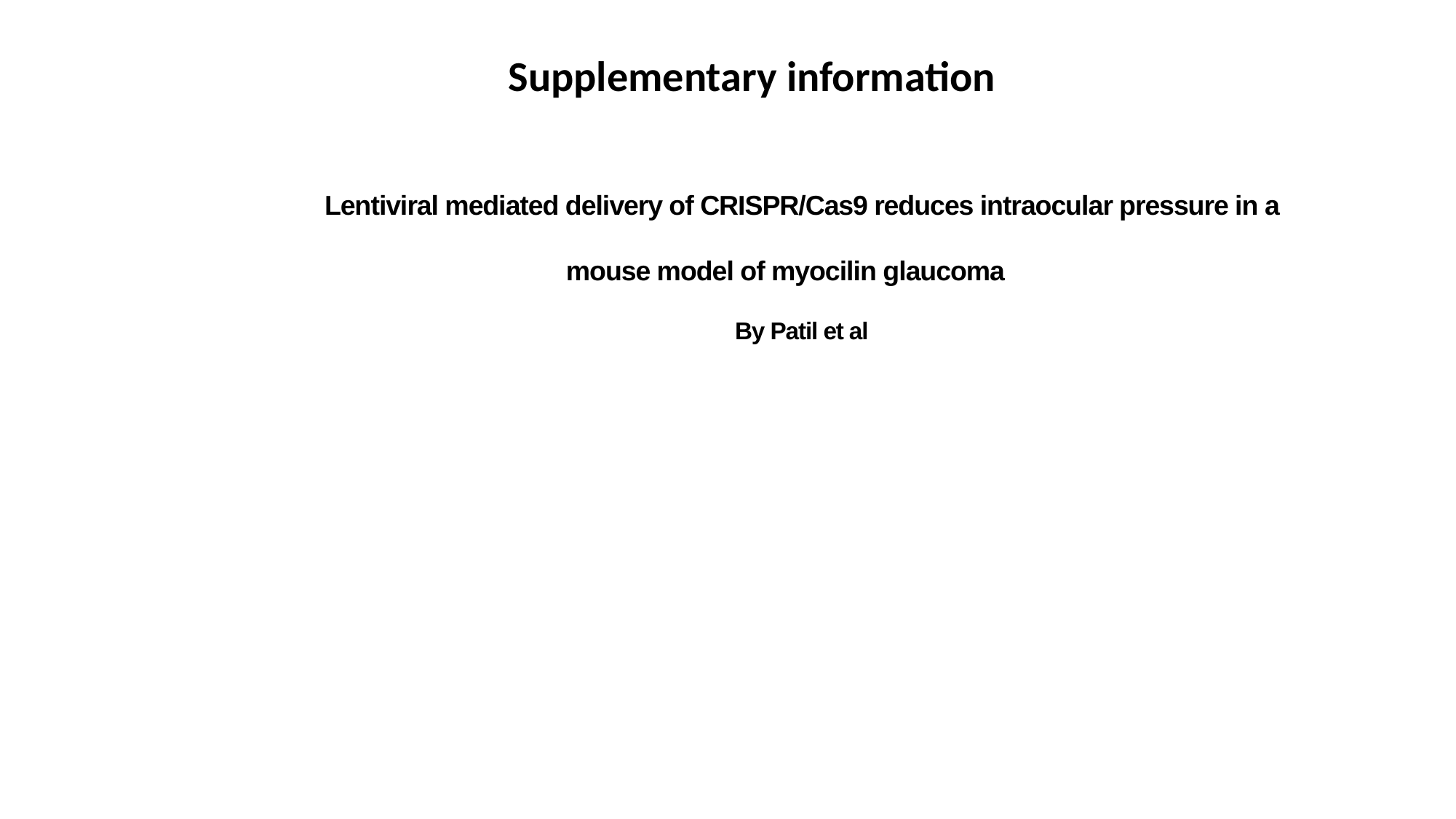

Supplementary information
Lentiviral mediated delivery of CRISPR/Cas9 reduces intraocular pressure in a mouse model of myocilin glaucoma
By Patil et al

## Slide 2
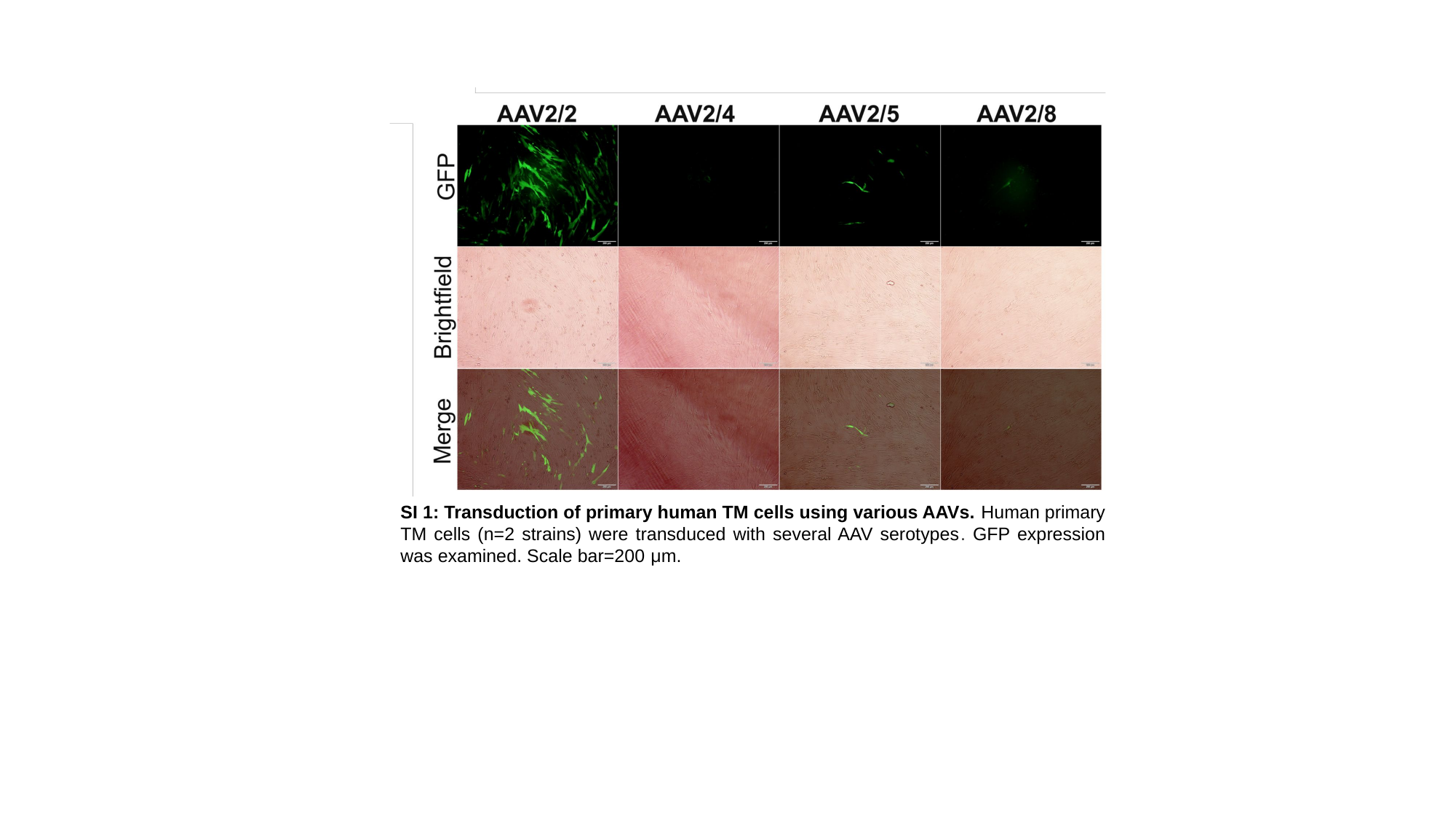

SI 1: Transduction of primary human TM cells using various AAVs. Human primary TM cells (n=2 strains) were transduced with several AAV serotypes. GFP expression was examined. Scale bar=200 μm.

## Slide 3
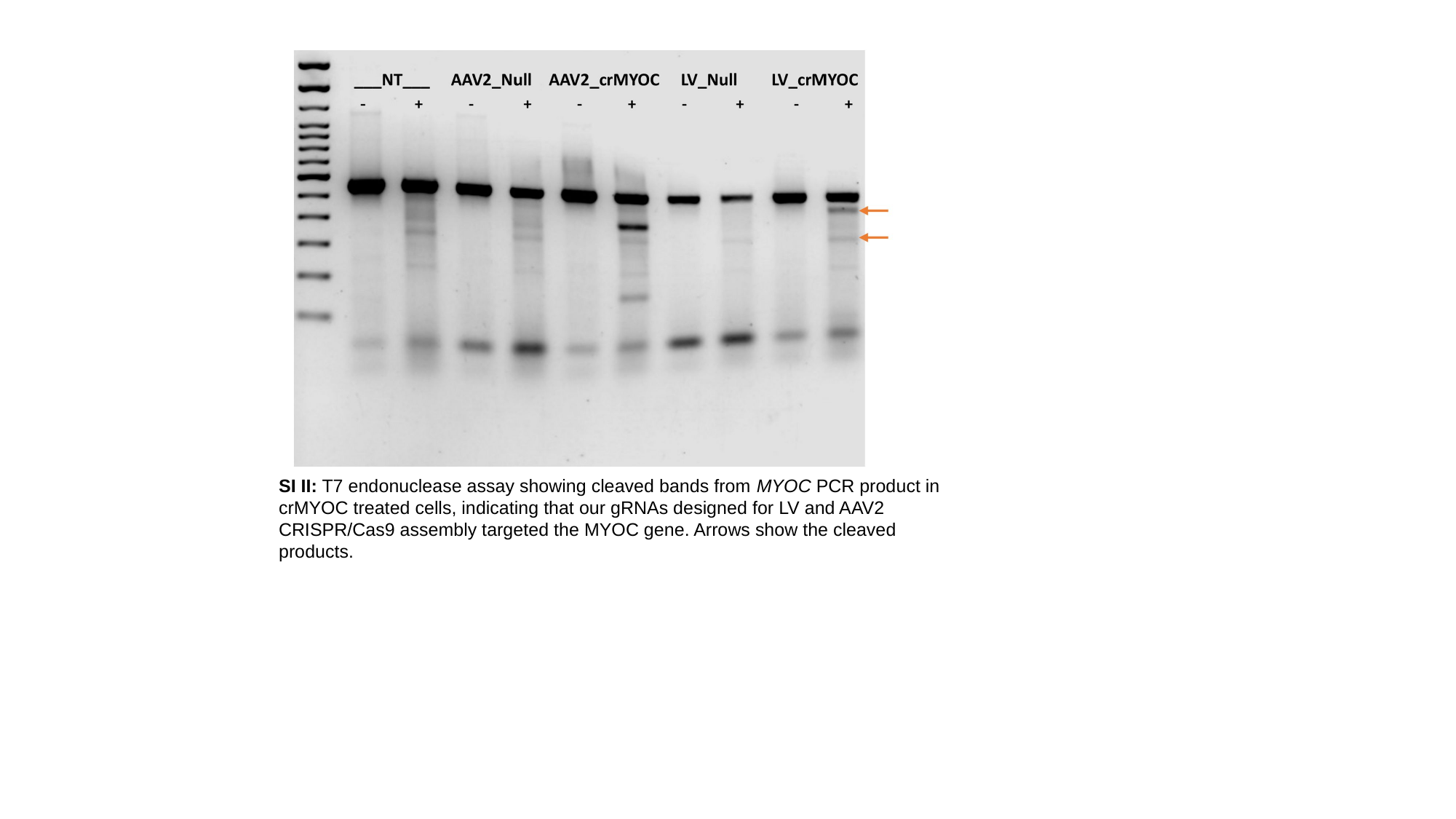

SI II: T7 endonuclease assay showing cleaved bands from MYOC PCR product in crMYOC treated cells, indicating that our gRNAs designed for LV and AAV2 CRISPR/Cas9 assembly targeted the MYOC gene. Arrows show the cleaved products.

## Slide 4
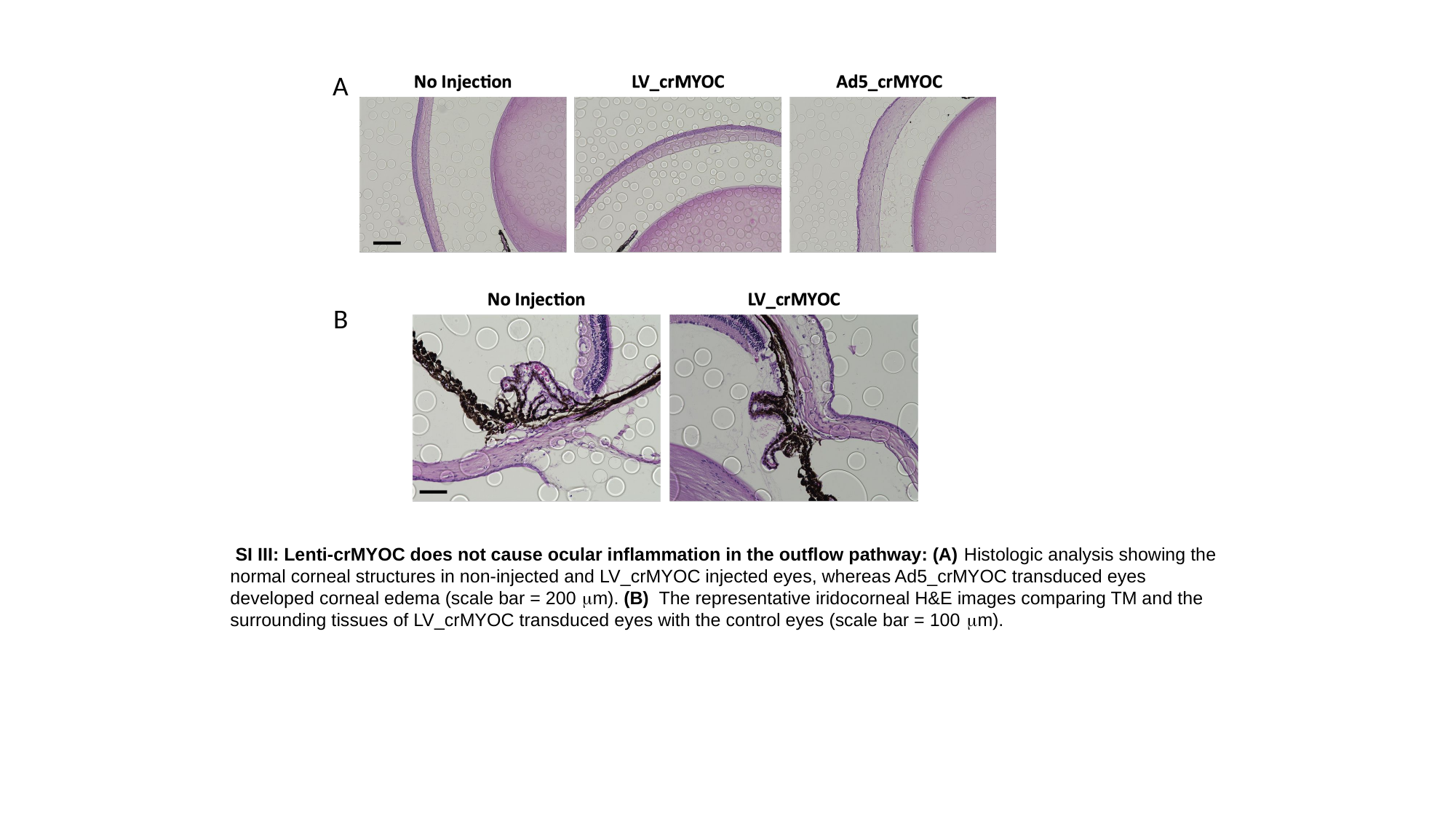

A
B
C
 SI III: Lenti-crMYOC does not cause ocular inflammation in the outflow pathway: (A) Histologic analysis showing the normal corneal structures in non-injected and LV_crMYOC injected eyes, whereas Ad5_crMYOC transduced eyes developed corneal edema (scale bar = 200 μm). (B) The representative iridocorneal H&E images comparing TM and the surrounding tissues of LV_crMYOC transduced eyes with the control eyes (scale bar = 100 μm).

## Slide 5
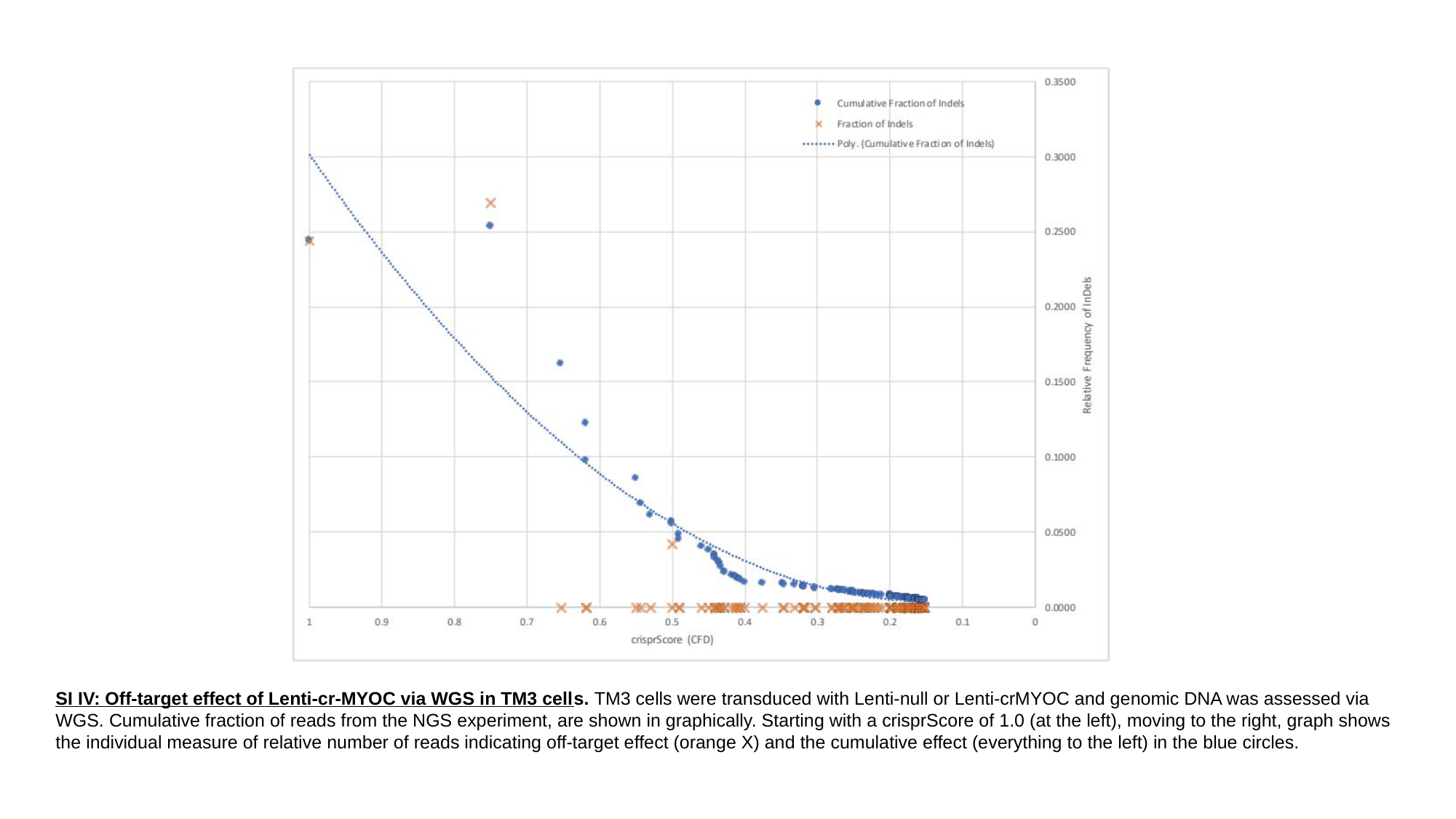

SI IV: Off-target effect of Lenti-cr-MYOC via WGS in TM3 cells. TM3 cells were transduced with Lenti-null or Lenti-crMYOC and genomic DNA was assessed via WGS. Cumulative fraction of reads from the NGS experiment, are shown in graphically. Starting with a crisprScore of 1.0 (at the left), moving to the right, graph shows the individual measure of relative number of reads indicating off-target effect (orange X) and the cumulative effect (everything to the left) in the blue circles.
