# Supplementary material for: Lentiviral mediated delivery of CRISPR/Cas9 reduces intraocular pressure in a mouse model of myocilin glaucoma
Source: Sci Rep. 2024 Mar 23;14:6958. doi: 10.1038/s41598-024-57286-6 (PMC10960846; doi:10.1038/s41598-024-57286-6)
Supplement: Supplementary file 3 — Raw data. [file 41598_2024_57286_MOESM3_ESM.pptx]

## Slide 1
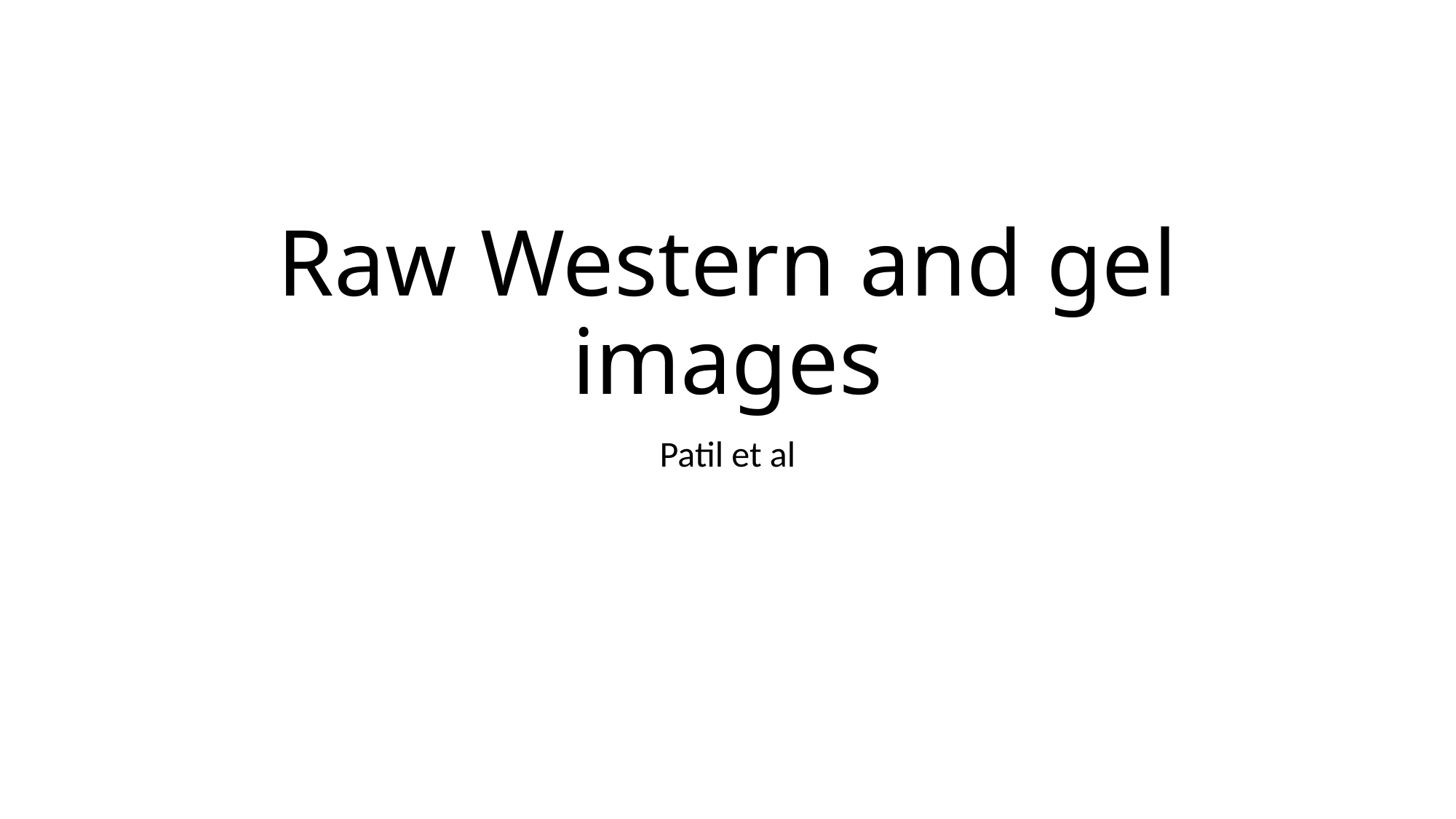

# Raw Western and gel images
Patil et al

## Slide 2
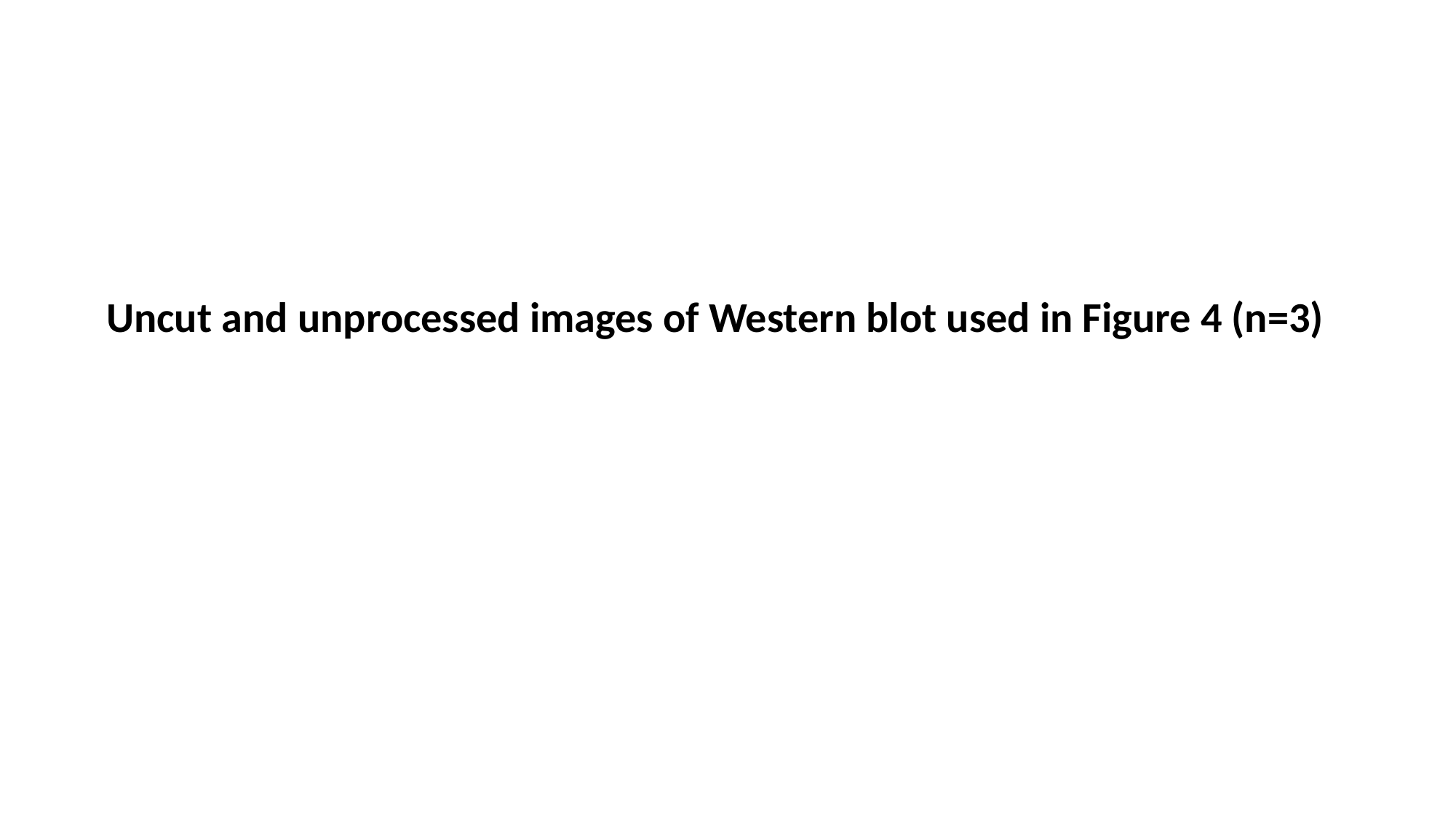

Uncut and unprocessed images of Western blot used in Figure 4 (n=3)

## Slide 3
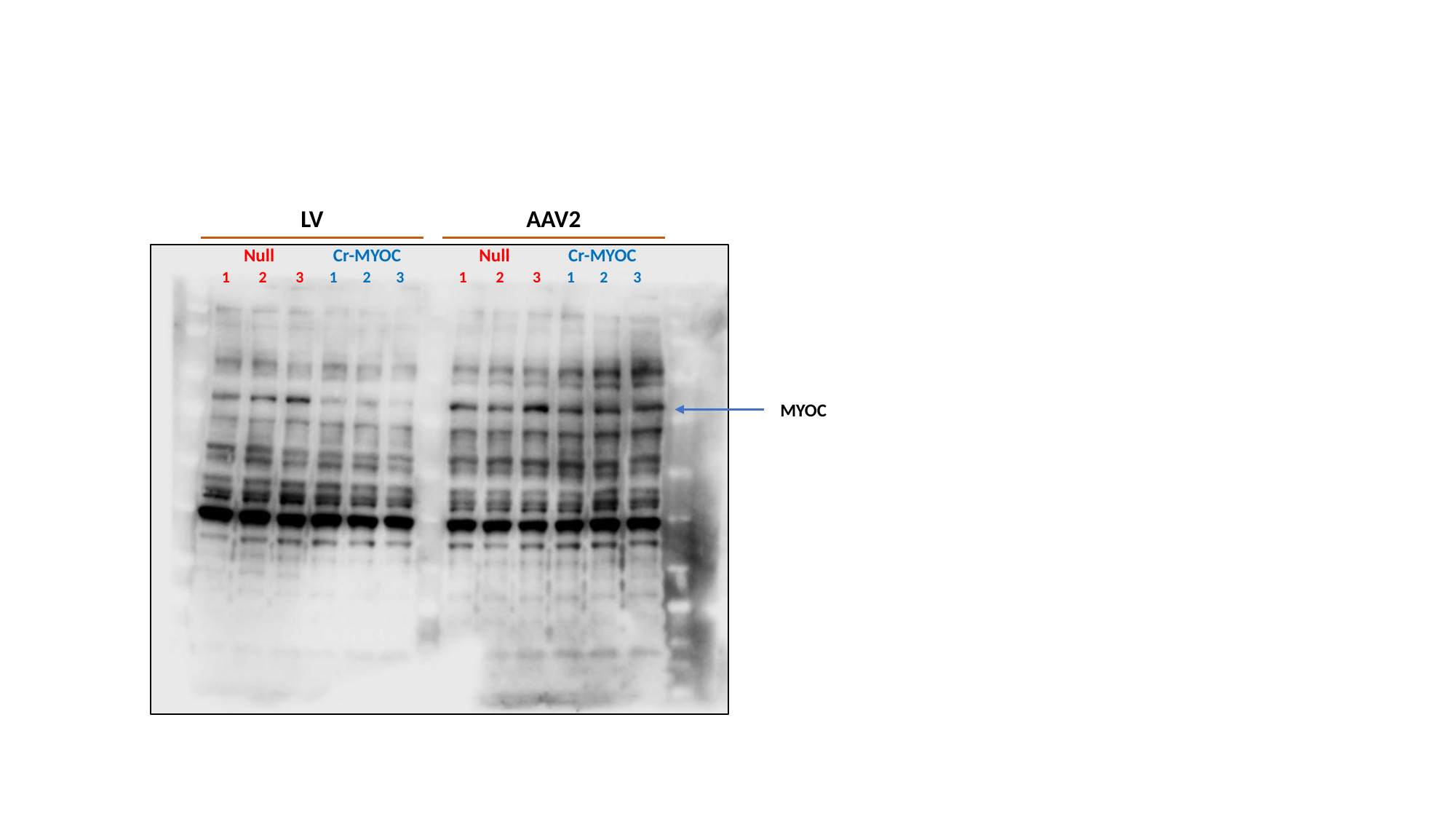

LV
AAV2
 Null Cr-MYOC
 Null Cr-MYOC
 1 2 3 1 2 3 1 2 3 1 2 3
MYOC

## Slide 4
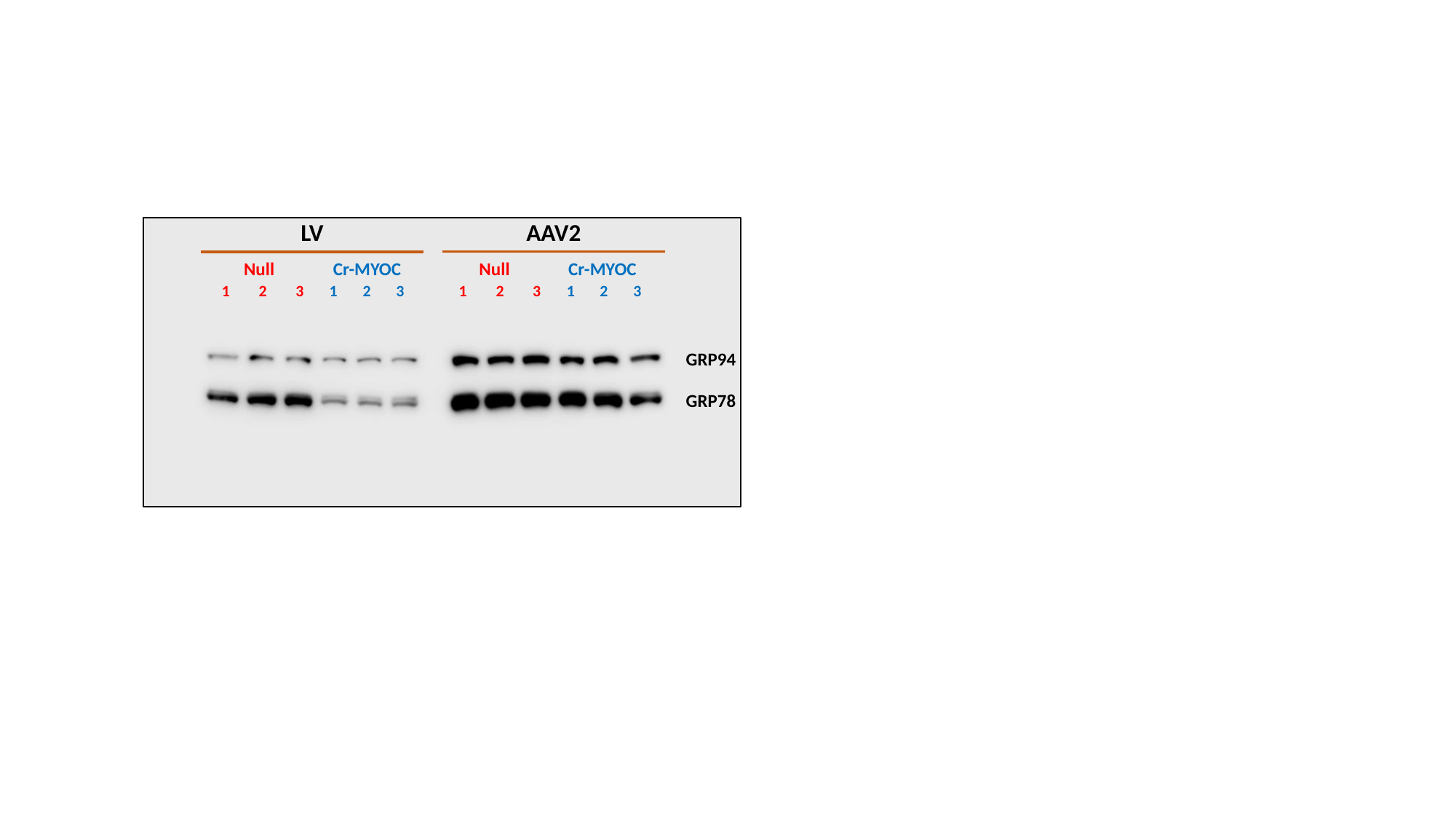

LV
AAV2
 Null Cr-MYOC
 Null Cr-MYOC
 1 2 3 1 2 3 1 2 3 1 2 3
GRP94
GRP78

## Slide 5
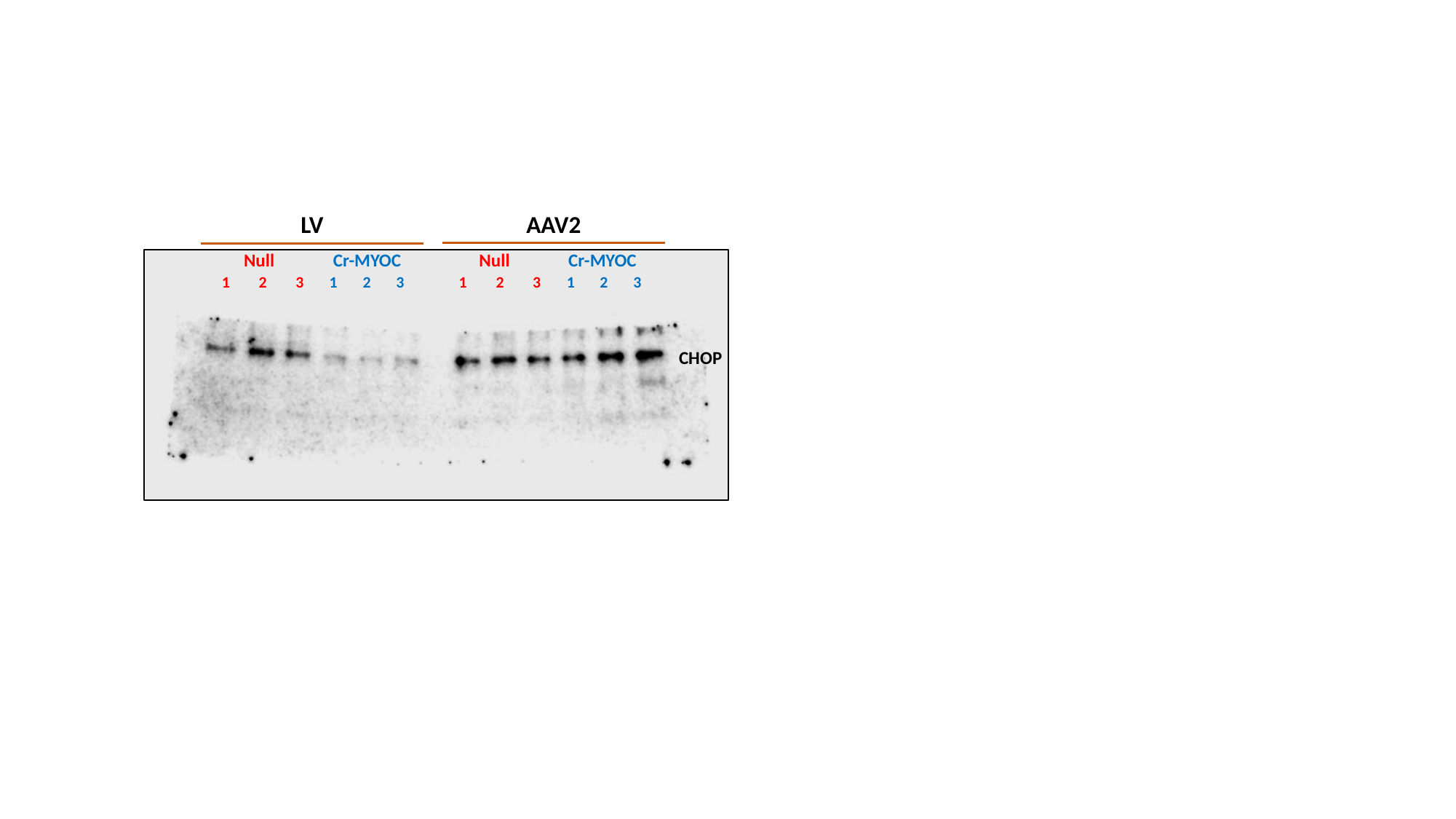

LV
AAV2
 Null Cr-MYOC
 Null Cr-MYOC
 1 2 3 1 2 3 1 2 3 1 2 3
CHOP

## Slide 6
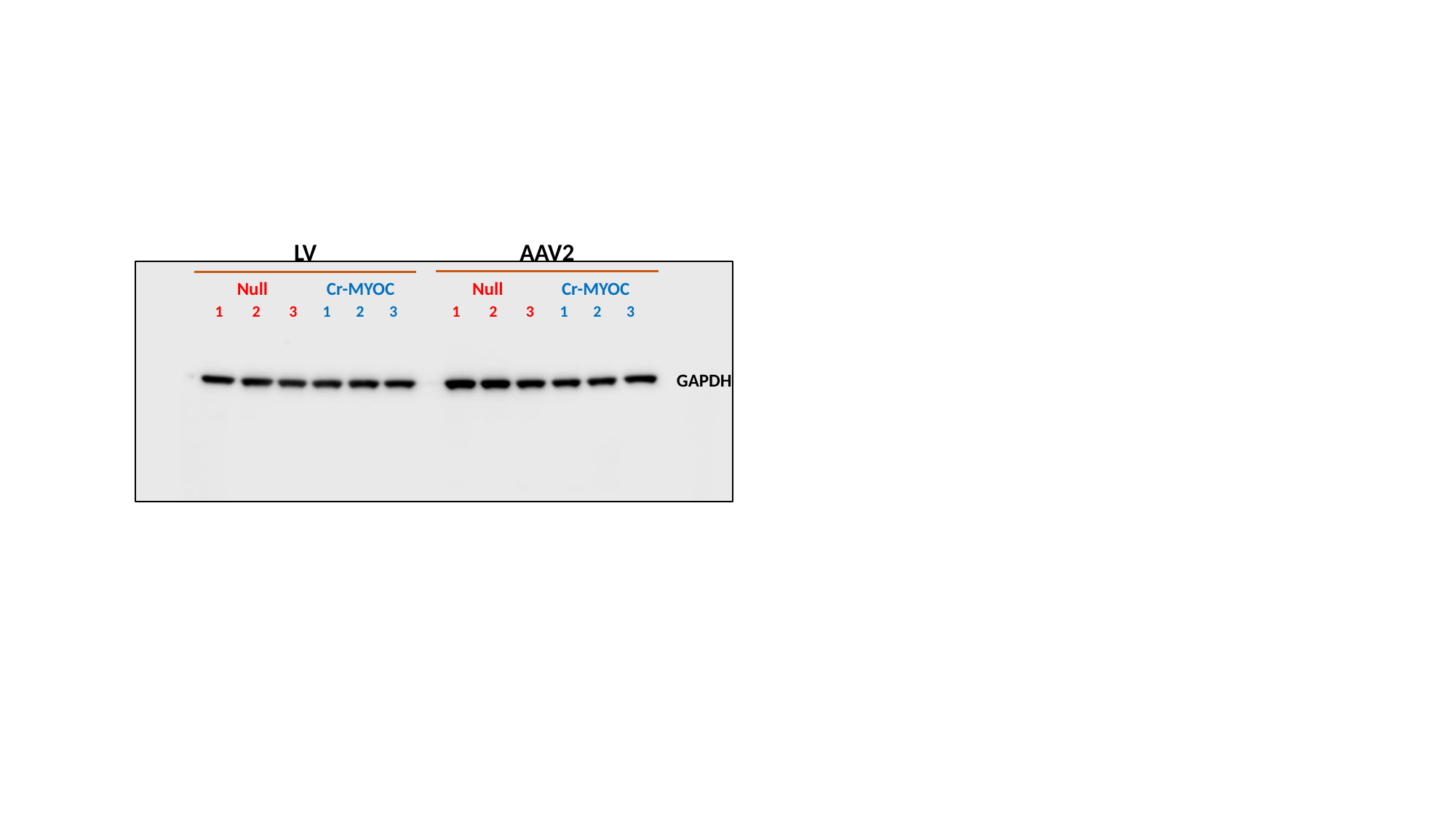

LV
AAV2
 Null Cr-MYOC
 Null Cr-MYOC
 1 2 3 1 2 3 1 2 3 1 2 3
GAPDH

## Slide 7
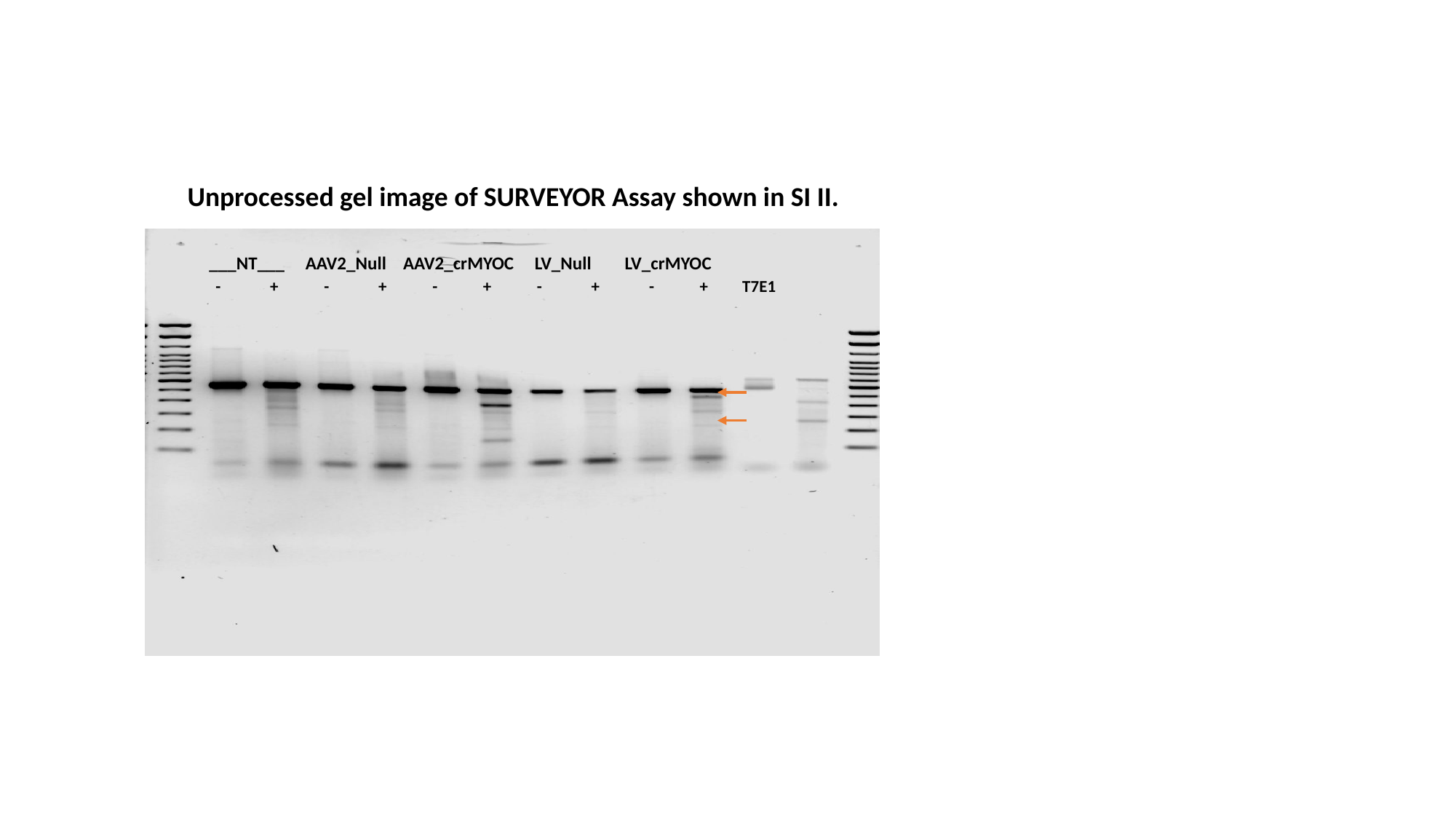

Unprocessed gel image of SURVEYOR Assay shown in SI II.
 ___NT___ AAV2_Null AAV2_crMYOC LV_Null LV_crMYOC
 - + - + - + - + - + T7E1
